# Systematic Screening of Trigger Moieties for Designing Formaldehyde Fluorescent Probes and Application in Live Cell Imaging

**DOI:** 10.3390/bios12100855

**Published:** 2022-10-10

**Authors:** Yin Jiang, Shumei Huang, Minghui Liu, Zejun Li, Weimin Xiao, Huatang Zhang, Liu Yang, Hongyan Sun

**Affiliations:** 1 School of Chemical Engineering and Light Industry, Guangdong University of Technology, Guangzhou 510006, China; 2 School of Biomedical and Pharmaceutical Sciences, Guangdong University of Technology, Guangzhou 510006, China; 3 Shenzhen Academy of Metrology & Quality Inspection, Shenzhen 518110, China; 4 College of Chemistry and Chemical Engineering, Central South University, Changsha 410083, China; 5 Department of Chemistry and Center of Super-Diamond and Advanced Films (COSDAF), City University of Hong Kong, 83 Tat Chee Avenue, Kowloon, Hong Kong 999077, China; 6 Key Laboratory of Biochip Technology, Biotech and Health Centre, Shenzhen Research Institute of City University of Hong Kong, Shenzhen 518057, China

**Keywords:** formaldehyde, fluorescent probe, 2−aza−Cope rearrangement, cell imaging

## Abstract

Formaldehyde (FA) is involved in multiple physiological regulatory processes and plays a crucial role in memory storage. Meanwhile, FA has a notorious reputation as a toxic compound, and it will cause a variety of diseases if its level is unbalanced in the human body. To date, there have been numerous fluorescent probes for FA imaging reported. Among them, the probes based on the 2−aza−Cope rearrangement have attracted the most attention, and their applications in cell imaging have been greatly expanded. Herein, we screened the various trigger moieties of FA fluorescent probes based on the mechanism of 2−aza−Cope rearrangement. FA−2, in which a fluorophore is connected to a 4−nitrobenzylamine group and an allyl group, demonstrated the highest sensitivity, selectivity, and reaction kinetics. Furthermore, FA−Lyso, derived from FA−2, has been successfully designed and applied to monitor exogenous and endogenous FA fluctuations in lysosomes of living cells.

## 1. Introduction

As the simplest aldehyde molecule, formaldehyde (FA) is used in many fields, including building materials, wood furniture, paint pigments, cosmetics, preservatives, and disinfectants in medical laboratories [1]. FA is also known as a hazardous substance because it can cause serious health problems. Nonetheless, FA can be generated endogenously via the demethylation of N−methylated amino acid residues by demethylase and oxidase enzymes [2]. As a result, FA maintains a concentration between 0.2–0.4 mM and acts as a metabolic intermediate in the normal physiological environment of the brain, which is essential for memory acquisition through the DNA demethylation cycle and cognitive function [3,4,5,6]. When FA is overloaded, it can cause Alzheimer’s disease (AD), diabetes, respiratory illness, cancer, and other diseases [7]. Consequently, developing robust tools to monitor FA concentration fluctuations in living cells and tissues is critical.

Due to its high sensitivity, selectivity, and spatial and temporal resolution, fluorescent techniques have attracted continuous attention and have been applied in the detection of many types of biomolecules in living organisms [8,9,10,11,12,13,14,15,16]. To date, two primary strategies have been reported for FA fluorescent probe design: one employs the 2−aza−Cope mechanism of rearrangement [17,18], and the other utilizes the condensation reaction mechanism between amines or hydrazones [19,20]. Among these design strategies, the method based on 2−aza−Cope mechanism has attracted much interest due to its high selectivity for FA. However, up to now, there has been a lack of systematic screening of trigger moieties based on the performance of selectivity, sensitivity, kinetics, etc. [21,22,23,24,25,26,27].

**Scheme 1:**
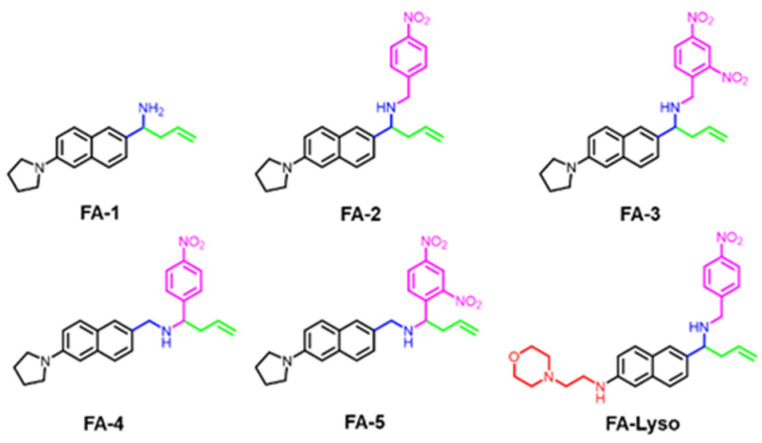
FA fluorescent probes with different reaction units.

Herein, we developed a number of fluorescent probes (**FA−1**, **FA−2**, **FA−3**, **FA−4**, **FA−5**) with different FA reaction units based on the same fluorescent dye to evaluate their overall efficacy of FA detection (Scheme 1). Under the same conditions, the detailed comparison study revealed that **FA−2**, in which the fluorophore is connected with a 4−nitrobenzylamine group and an allyl group on the carbonyl carbon atom, demonstrated the highest sensitivity, selectivity, and reaction kinetics. On the basis of **FA−2**, **FA−Lyso** has been further designed and applied to monitor exogenous and endogenous FA fluctuations in lysosomes in living cells successfully.

## 2. Materials and Methods

### 2.1. Materials and Instruments

#### 2.1.1. Apparatus

^1^H NMR and ^13^C NMR spectra were obtained on a Bruker 400 MHz spectrometer (Bruker BioSpin, Fällanden, Switzerland). Mass spectrometry was performed with Thermo TSQ Endura Triple Quadrupole Mass Spectrometer (Thermo Fisher, Frederick, MD, USA). UV absorption spectra were recorded on Shimadzu UV−3600 Plus UV−VIS−NIR Spectrophotometer (Shimadzu Corporation, Kyoto, Japan). Fluorescence spectra were acquired with a FluoroMax−4 fluorescence photometer (Horiba Scientific, Kyoto, Japan). Cell imaging was performed by a ZEISS LSM 800 Confocal Laser Scanning (Carl Zeiss AG, Oberkochen, Germany) Microscope. Milli−Q water was applied in all experiments.

#### 2.1.2. Reagents

All reagents were purchased from commercial suppliers and used as received unless otherwise noted. 6−hydroxy−2−naph−thaldehyde, trifluoromethanesulfonic anhydride, allylboronic acid pinacol ester, 4−(2−aminoethyl) morpholine, BINAP, and sodium borohydride were bought from Bidepharm (Bide Pharmatech Co., Ltd., Shanghai, China). Pyrrolidine and NH_3_ (7 M in methanol) were purchased from J&K Chemical (J&K Chemical Ltd., Beijing, China). Cs_2_CO_3_, palladium (II) acetate, 2,4−dinitrobenzaldehyde, 4−nitrobenzaldehyde, anhydrous DCM, and methanol were obtained from Adamas−beta^®^ (Titan Scitific Co. Ltd., Shanghai, China). Milli−Q water was applied for the experiment and the preparation of all the buffer.

### 2.2. Synthesis

**FA−1**, **FA−2**, **FA−3**, **FA−4**, **FA−5**, and **FA−Lyso** were synthesized according to the route illustrated in Scheme S1 with satisfied reaction yields. Compounds A, B, C, and D were reported by our group or other research groups and synthesized as in the literature accordingly [24,28,29,30]. All the characterizations of products were performed by ^1^H NMR, ^13^C NMR, and ESI−MS and shown in Supplementary Materials.

Synthesis of compound E

Palladium (II) acetate (23 mg, 0.102 mmol) and BINAP (54 mg, 0.87 mmol) were added into the mixture of compound D (500 mg, 1.7 mmol), 4−(2−aminoethyl) morpholine (1140 μL, 8.7 mmol) and cesium carbonate (848 mg, 2.6 mmol) in toluene under N_2_ and stirred at 110 °C for 4 h. After that, the reaction was cooled to room temperature and water and ethyl acetate were added for extraction. Then, the combined organic phase was washed with saturated NaCl, dried over MgSO_4_, filtered, and concentrated. Compound E was purified by flash chromatography on silica (petroleum ether: ethyl acetate = 1:1) as light−yellow solid (243 mg, 49%). ^1^H NMR (400 MHz, CDCl_3_) δ 10.00 (s, 1H), 8.12 (s, 1H), 7.82 (dd, *J* = 8.5, 1.7 Hz, 1H), 7.75 (d, *J* = 8.8 Hz, 1H), 7.63 (d, *J* = 8.6 Hz, 1H), 6.97 (dd, *J* = 8.8, 2.4 Hz, 1H), 6.78 (d, *J* = 2.3 Hz, 1H), 4.87 (t, *J* = 4.9 Hz, 1H), 3.74 (t, *J* = 4.6 Hz, 4H), 3.31 (q, *J* = 5.1 Hz, 2H), 2.71 (t, *J* = 6.8 Hz, 2H), 2.51 (t, *J* = 4.44 Hz, 4H). ^13^C NMR (100 MHz, CDCl_3_) δ 192.03, 148.95, 139.23, 134.80, 130.96, 130.82, 126.72, 126.18, 123.96, 118.89, 104.00, 67.07, 56.82, 53.42, 39.48.

Synthesis of compound F

NH_3_ solution (300 µL, 7.0 N in CH_3_OH, 18 mmol) was added into the solution of compound A (500 mg, 1.8 mmol) in anhydrous dichloromethane (DCM) in an ice bath under N_2_. After stirring for 30 min, allylboronic acid pinacol ester (411 µL, 2 mmol) was added and the solution was warmed to room temperature and stirred overnight. Then, the solvent was removed under reduced pressure, and the crude product was further purified by silica column chromatography to gain the compound F (91 mg, 15%). ^1^H NMR (400 MHz, CDCl_3_) δ 7.63 (d, *J* = 3.28 Hz, 2H), 7.60 (d, *J* = 2.76 Hz, 1H), 7.37 (dd, *J* = 8.48, 1.68 Hz, 1H), 6.93 (dd, *J* = 8.80, 2.32 Hz, 1H), 6.80 (d, *J* = 2.12 Hz, 1H), 5.78−5.88 (m, 1H), 5.17 (d, J = 17.2 Hz, 1H), 5.13 (d, J = 11.1 Hz, 1H), 4.83 (t, *J* = 6.56 Hz, 1H), 3.74 (m, 4H), 3.28 (t, *J* = 5.76 Hz, 2H), 2.7 (t, *J* = 6.04 Hz, 2H), 2.59 (t, J = 7.1 Hz, 2H), 2.51 (m, 4H). ^13^C NMR (100 MHz, CDCl_3_) δ 146.38, 134.93, 134.36, 129.12, 127.35, 126.55, 125.69, 125.30, 118.54, 118.50, 104.38, 67.08, 57.13, 55.75, 53.50, 42.20, 40.05.

Synthesis of **FA−1**

NH_3_ solution (370 µL, 7.0 M in MeOH, 22 mmol) was added into the solution of compound A (500 mg, 2.2 mmol) in anhydrous DCM in an ice bath under N_2_. After stirring for 30 min, allylboronic acid pinacol ester (502 µL, 2.4 mmol) was added and the solution was warmed to room temperature and stirred overnight. The solvent was removed under reduced pressure, and the crude product was further purified by silica column chromatography to gain the product **FA−1** (112 mg, 19% yield). ^1^H NMR (400 MHz, CDCl_3_) δ 7.66 (d, *J* = 8.9 Hz, 1H), 7.61 (d, *J* = 4.0 Hz, 1H), 7.60 (d, *J* = 3.5 Hz, 1H), 7.34 (dd, *J* = 8.6, 1.6 Hz, 1H), 6.98 (dd, *J* = 8.9, 2.4 Hz, 1H), 6.73 (d, *J* = 2.2 Hz, 1H), 5.80–5.70 (m, 1H), 5.14–5.10 (d, *J* = 17.1 Hz, 1H), 5.07–5.04 (d, *J* = 10.2 Hz, 1H), 4.11–4.07 (m, 1H), 3.39 (m, 4H), 2.58–2.46 (m, 2H), 2.33 (s, 2H), 2.06–2.03 (m, 4H). ^13^C NMR (100 MHz, CDCl_3_) δ 146.00, 138.47, 135.89, 134.66, 128.81, 126.19, 125.24, 124.62, 117.54, 115.99, 104.78, 55.58, 47.98, 44.15, 25.61. ESI−MS: calcd. for C_18_H_23_N_2_ [M + H]^+^ 267.1861, found 267.1850.

Synthesis of the probe **FA−2** and **FA−3**

**FA−1** (1 eq) and 4−nitrobenzaldehyde/2,4−dinitrobenzaldehyde (1.2 eq) were dissolved in chloroform and stirred at room temperature for 3 h. Then, MeOH was added as solvent and NaBH_4_ (10 eq) was added into the mixture in batches within 3 h at 0 °C. After stirring overnight at 0 °C, the solvent was removed by reduced pressure and the residue was separated by silica gel column to provide the products.

**FA−2** (53% yield). ^1^H NMR (400 MHz, CDCl_3_) δ 8.14 (d, *J* = 8.7 Hz, 2H), 7.66 (d, *J* = 9.0 Hz, 1H), 7.63 (d, *J* = 8.6 Hz, 1H), 7.54 (s, 1H), 7.42 (d, *J* = 8.7 Hz, 2H), 7.35 (dd, *J* = 8.5, 1.7 Hz, 1H), 7.00 (dd, *J* = 8.9, 2.4 Hz, 1H), 6.76 (d, *J* = 2.1 Hz, 1H), 5.83–5.68 (m, 1H), 5.11 (d, *J* = 17.1 Hz, 1H), 5.05 (d, *J* = 10.1 Hz, 1H), 3.77 (d, *J* = 14.4 Hz, 1H), 3.73 (d, *J* = 6.2 Hz, 1H), 3.68 (d, *J* = 14.5 Hz, 1H), 3.40 (t, *J* = 6.6 Hz, 4H), 2.56–2.42 (m, 2H), 2.09–2.03 (m, 4H). ^13^C NMR (100 MHz, CDCl_3_) δ 148.86, 147.06, 146.13, 135.69, 134.94, 128.84, 128.74, 126.45, 126.22, 126.15, 125.45, 123.65, 117.69, 116.07, 104.82, 62.04, 50.76, 48.02, 43.03, 25.63. ESI−MS: calcd. for C_25_H_28_N_3_O_2_ [M + H]^+^ 402.2182, found 402.2179.

**FA−3** (49% yield). ^1^H NMR (400 MHz, CDCl_3_) δ 8.67 (d, *J* = 2.3 Hz, 1H), 8.23 (dd, *J* = 8.5, 2.3 Hz, 1H), 7.68 (d, *J* = 8.5 Hz, 1H), 7.62 (d, *J* = 8.9 Hz, 1H), 7.56 (d, *J* = 8.5 Hz, 1H), 7.48 (s, 1H), 7.28 (dd, *J* = 8.08, 1.68 Hz, 1H), 6.98 (dd, *J* = 8.9, 2.4 Hz, 1H), 6.71 (d, *J* = 2.2 Hz, 1H), 5.83–5.67 (m, 1H), 5.11 (d, *J* = 17.2 Hz, 1H), 5.07 (d, *J* = 10.2 Hz, 1H), 4.06 (d, *J* = 15.5 Hz, 1H), 3.95 (d, *J* = 15.5 Hz, 1H), 3.73 (dd, *J* = 7.8, 5.9 Hz, 1H), 3.39 (t, *J* = 6.6 Hz, 4H), 2.55–2.41 (m, 2H), 2.08–2.03 (m, 4H). ^13^C NMR (100 MHz, CDCl_3_) δ 148.95, 146.63, 146.16, 143.54, 135.34, 135.13, 134.99, 132.76, 128.71, 126.80, 126.46, 126.36, 125.99, 125.39, 120.09, 117.96, 116.11, 104.71, 62.75, 48.47, 47.98, 42.83, 25.62. ESI−MS: calcd. for C_25_H_27_N_4_O_4_ [M + H]^+^ 447.2032, found 447.2027.

Synthesis of the probe **FA−4** and **FA−5**

Compound A (1.2 eq) and compound B/C (1 eq) were dissolved in CHCl_3_ and stirred at room temperature for 3 h. Then, MeOH was added as solvent and NaBH_4_ (10 eq) was added into the mixture in batches within 3 h at 0 °C. After stirring overnight at 0 °C, the solvent was removed by reduced pressure, and the residue was separated by silica gel column to provide the products.

**FA−4** (53% yield). ^1^H NMR (400 MHz, CDCl_3_) δ 8.22 (d, *J* = 8.8 Hz, 2H), 7.64 (d, *J* = 8.9 Hz, 1H), 7.60 (d, *J* = 8.5 Hz, 1H), 7.56 (d, *J* = 8.7 Hz, 2H), 7.45 (s, 1H), 7.23 (dd, *J* = 8.4, 1.7 Hz, 1H), 7.00 (dd, *J* = 8.9, 2.4 Hz, 1H), 6.75 (d, *J* = 2.2 Hz, 1H), 5.73–5.61 (m, 1H), 5.09 (s, 1H), 5.07–5.04 (m, 1H), 3.87–3.82 (m, 1H), 3.76 (d, *J* = 13.2 Hz, 1H), 3.58 (d, *J* = 13.2 Hz, 1H), 3.40 (t, *J* = 6.6 Hz, 4H), 2.43–2.36 (m, 2H), 2.09–2.03 (m, 4H). ^13^C NMR (100 MHz, CDCl_3_) δ 152.15, 147.28, 146.05, 134.64, 134.46, 132.49, 128.67, 128.34, 126.87, 126.58, 126.26, 126.12, 123.82, 118.61, 116.05, 104.76, 61.08, 51.88, 47.96, 43.04, 25.62. ESI−MS: calcd. for C_25_H_28_N_3_O_2_ [M + H]^+^ 402.2181, found 447.2177.

**FA−5** (49% yield). ^1^H NMR (400 MHz, CDCl_3_) δ 8.58 (d, *J* = 2.3 Hz, 1H), 8.35 (dd, *J* = 8.7, 2.3 Hz, 1H), 8.20 (d, *J* = 8.7 Hz, 1H), 7.58 (d, *J* = 8.9 Hz, 1H), 7.53 (d, *J* = 8.4 Hz, 1H), 7.35 (s, 1H), 7.15 (dd, *J* = 8.4, 1.5 Hz, 1H), 6.97 (dd, *J* = 8.9, 2.4 Hz, 1H), 6.69 (d, *J* = 2.1 Hz, 1H), 5.82–5.71 (m, 1H), 5.15 (d, *J* = 5.9 Hz, 1H), 5.11 (d, *J* = 13.8 Hz, 1H), 4.36 (dd, *J* = 8.8, 4.1 Hz, 1H), 3.63 (s, 2H), 3.39 (t, *J* = 6.5 Hz, 4H), 2.58–2.64(m, 1H), 2.25–2.33 (m, 1H), 2.08–2.02 (m, 4H). ^13^C NMR (100 MHz, CDCl_3_) δ 149.61, 146.75, 146.44, 146.13, 134.68, 133.91, 131.22, 128.67, 126.86, 126.83, 126.35, 125.93, 119.60, 119.47, 116.12, 104.69, 56.93, 52.84, 47.94, 42.30, 29.84, 25.64. ESI−MS: calcd. for C_25_H_27_N_4_O_4_ [M + H]^+^ 447.2032, found 447.2021.

Synthesis of the probe **FA−Lyso**

Compound F (90 mg, 0.3 mmol) and 4−nitrobenzaldehyde (55 mg, 0.36 mmol) were dissolved in chloroform and stirred at room temperature for 3 h. Then, MeOH was added as solvent and NaBH_4_ (113.2 mg, 3 mmol) was added into the mixture in batches within 3 h at 0 °C. After stirring overnight at 0 °C, the solvent was removed by reduced pressure, and the residue was chromatographed on a silica gel column to provide the probe **FA−Lyso** (56 mg, 40%). ^1^H NMR (400 MHz, CDCl_3_) δ 8.14 (d, *J* = 8.6 Hz, 2H), 7.63 (d, *J* = 5.7 Hz, 1H), 7.60 (d, *J* = 6.0 Hz, 1H), 7.55 (s, 1H), 7.42 (d, *J* = 8.5 Hz, 2H), 7.37 (d, *J* = 8.4 Hz, 1H), 6.95 (dd, *J* = 8.8, 2.1 Hz, 1H), 6.82 (s, 1H), 5.83–5.65 (m, 1H), 5.11 (d, *J* = 17.1 Hz, 1H), 5.06 (d, *J* = 10.2 Hz, 1H), 3.79–3.72 (m, 6H), 3.67 (d, *J* = 14.4 Hz, 1H), 3.28 (t, *J* = 5.8 Hz, 2H), 2.70 (t, *J* = 5.9 Hz, 2H), 2.56–2.43 (m, 6H). ^13^C NMR (100 MHz, CDCl_3_) δ 148.77, 147.04, 146.29, 136.54, 135.57, 134.89, 128.85, 128.82, 127.42, 126.54, 126.28, 125.64, 123.67, 118.46, 117.84, 104.50, 67.13, 61.96, 57.13, 53.50, 50.78, 43.07, 40.14. ESI−MS: calcd. for C_27_H_33_N_4_O_3_ [M + H]^+^ 461.2553, found 461.2550.

### 2.3. General Procedure for Absorption and Fluorescent Measurement

Firstly, 5 mM stock solution of probe **FA−1**~**FA−5** were prepared in appropriate DMSO. Cys, Hcy, and GSH were dissolved in PBS buffer (pH 7.4, 50 mM) to form 500 mM stock solution. CaCl_2_, ZnCl_2_, FeCl_2_, FeCl_3_, CuCl_2_, KNO_3_, NaNO_2_, NaHSO_3_, Na_2_SO_3_, and NaSH were prepared as 500 mM stock solution in water. Glyoxal, 4−hydroxybenzaldehyde, benzaldehyde, crotonaldehyde, and 4−nitrobenzaldehyde were prepared as 500 mM stock solution in DMSO. Concentrations of H_2_O_2_ and HOCl were detected by UV absorption experiment by Beer–Lambert Law. Specifically, the extinction coefficient (ε) of −OCl and H_2_O_2_ were 350 M^−1^ cm^−1^ and 43.6 M^−1^ cm^−1^ as well as the determined absorption was at 292 nm and 240 nm, respectively [31,32].

The stock solution of probe **FA−1**, **FA−2**, **FA−3**, **FA−4**, and **FA−5** were added into a 5 mL volumetric flask of PBS solution (pH 5, 10 mM) and diluted to 5 μM as the final concentration. The different analytic species were then added, and the resulting solutions were incubated for 60 min at 37 °C. After that, the absorption and fluorescence experiments were conducted.

### 2.4. Cells Imaging

WI−38 cells were cultured in DMEM medium with 10% FBS and 1% antibiotics (penicillin and streptomycin) at 37 °C under 5% CO_2_ atmosphere and seeded in a glass−bottomed confocal dish (35 mm). After 24 h, NaHSO_3_ pretreated WI−38 cells were incubated with probe **FA−2** for 30 min. Then, FA was added into the medium for 1 h incubation. After washing with DPBS, the dishes were placed on a confocal microscope and fluorescence images were taken.

For colocalization imaging, the cells were treated with **FA−Lyso** for 30 min incubation firstly. Then, FA was added into the medium for another 30 min incubation. After that, cells were incubated with Lyso−Tracker Red and Mito−Tracker Deep Red for 15 min respectively. A confocal fluorescence image was taken of the dishes in the end.

The WI38 cell line and HeLa cell line were obtained from Dr. Yongguang Jia in SCUT.

## 3. Result and Discussion

To begin with, the fluorophore of 6−(pyrrolidine−1−yl)−2−naphthaldehyde (**FA-A**) was chosen as the basic building block due to its extraordinary features, including high quantum yield, facile modification, and moderate stability towards ROS, RNS, and RSS. Meanwhile, five recognition groups for FA were introduced into **FA-A**. All of the probes were synthesized successfully with satisfied yields. The synthesis routes and chemical structure characterizations (^1^H NMR, ^13^C NMR, and ESI−MS) were shown in Scheme S1 (Supplementary Materials).

After synthesis and characterization, initial absorption and fluorescence emission experiments were performed. The absorbance variation of all probes in the presence and absence of FA can be observed via UV−vis absorption spectrum (Figure S1 in Supplementary Materials). As a result of reaction with FA, **FA−1**, **FA−2**, and **FA−3** showed a notable enhancement and slight red−shift from 360 nm to 395 nm. However, **FA−4** exhibited just a mild increase in the presence of FA. As to **FA−5**, a remarkable and broad enhanced absorbance has been observed after the reaction with FA. Using the absorption results, we can conclude that all the probes showed the feasibility to react with FA in PBS buffer. This result strengthened our confidence in subsequent studies of the fluorescence performance.

**Figure 1:**
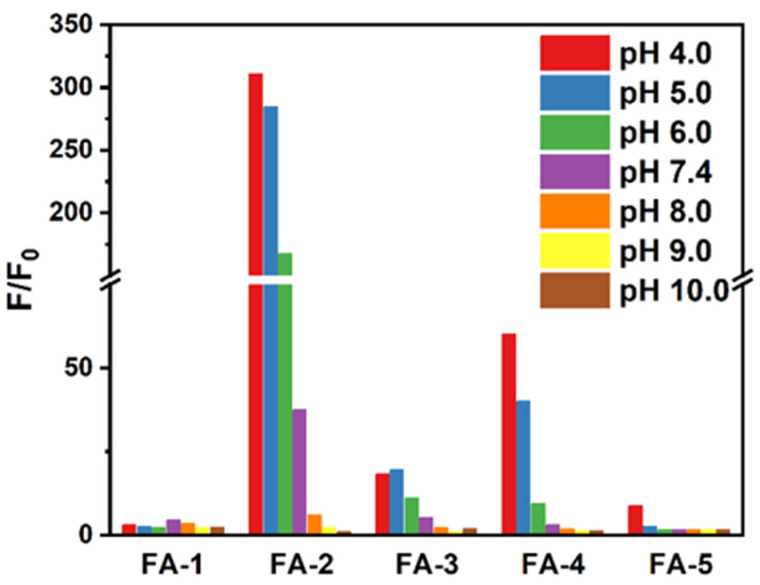
Systematic comparison of the response performance of five probes for FA sensing. Fluorescence intensity ratio (F/F_0_) of probes (**FA−1**–**FA−5**) before and after FA treatment in the pH range of 4−10; 5 μM probes were incubated with 2 mM FA in the experiment; For **FA−1**, data was collected at 442 and 530 nm correspondingly due to the ratiometric property of **FA−1**. For **FA−2** and **FA−3**, data was collected at 530 nm. For **FA−4** and **FA−5**, data was collected at 442 nm.

Next, we systematically investigated the reaction behavior of all probes towards FA by time−dependent fluorescence profiles under the different pH values. As shown in Figure 1 and Figure S2, **FA−1** itself possessed remarkable fluorescence and the maximum emission wavelength at 442 nm. After treatment with FA, the fluorescence signal at 442 nm declined, and a new emission at 530 nm occurred simultaneously. Despite that **FA−1** exhibited an increased ratio with the reaction of FA and a relatively optimal value at pH 7, the increased ratio was relatively low (<6−fold), which limited its application as the fluorescent probe (Figure 1 and Figure S2 in Supplementary Materials). For **FA−2**, the reaction kinetics were significantly fast, and the fluorescence at 530 nm was sharply increased (ca. 280−fold) in 1 h at pH 4 and 5 (Figure 1). The fluorescence response of **FA−2** with FA increased when pH value decreased, whereas no fluorescence enhancement was observed between pH 8–10 (Figure S3 in Supplementary Materials). **FA−3** also showed a similar response at 530 nm from pH 4 to 10 (Figure S4 in Supplementary Materials). Unlike **FA−2**, however, the fluorescence of **FA−3** increased by a much lower factor (Figure 1). For **FA−4**, the strong fluorescence signal was observed at around 442 nm after treating with FA under pH 4 and 5 (Figure 1, Figure S5 in Supplementary Materials). Similar fluorescence responses were obtained with **FA−5** and **FA−4** at the same pH value (Figure 1, Figure S6 in Supplementary Materials).

The obvious difference on maximum emission wavelength between **FA−2** and **FA−4** is due to the difference of reaction products. The reaction of **FA−2** and FA produces **FA-A**, which contains aldehyde compound as the electron−withdrawing group in the fluorophore (Scheme S2 in Supplementary Materials), while **FA−4** produces 4−nitrobenzaldehyde and **FA-P**, which is an alkylamine compound (Scheme S3 in Supplementary Materials) [17,24]. As the literature reported, higher electron−withdrawing effect in ICT fluorophore induces the redshift of emission wavelength, as well as the increase of fluorescence quantum yield [28,33]. To confirm the above theory, we synthesized fluorophore **FA-A** and **FA-P** and tested the fluorescence intensity in PBS buffer (pH 5, 10 mM). The result showed that **FA-P**, the reaction product of **FA−4**/**FA−5** with FA displayed the maximum emission wavelength at 442 nm with the fluorescent quantum yield of 0.11. However, the maximum emission wavelength of **FA-A**, which is the product of **FA−2**/**FA−3** and FA, is red−shifted to 530 nm with the fluorescent quantum yield of 0.25 (Figure S7 in Supplementary Materials) [22,28]. These results confirmed that **FA−2′**s fluorescence response is extraordinary for FA probe design based on the mechanism of 2−aza−Cope rearrangement.

**Figure 2:**
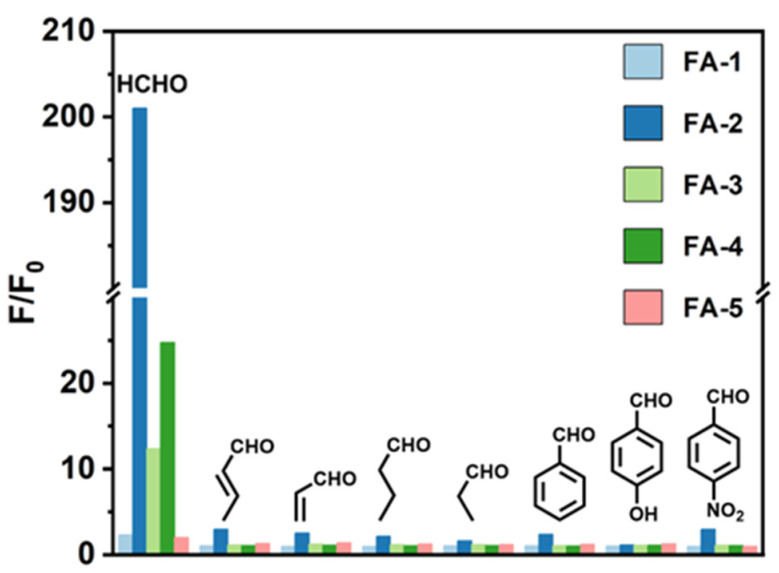
Fluorescence intensity ratio (F/F_0_) of probes (**FA−1**–**FA−5**, 5 μM) toward FA (500 μM) and various aldehyde compounds (500 μM). For **FA−1**, F/F_0_ is the fluorescence intensity ratio of I_530_/I_442_. For **FA−2**–**FA−5**, the fluorescent intensity at maximum wavelength was used.

To further compare the integrated capability towards FA detection, the selectivity experiment was carried out in PBS buffer (pH 5.0, 10 mM). The probes were treated with different biological species such as H_2_O_2_, HOCl, GSH, Hcy, Cys, Ca^2+^, Fe^2+^, Fe^3+^, etc. In addition, we also included several aldehyde compounds, crotonaldehyde, acraldehyde, butyraldehyde, propionaldehyde, benzaldehyde, *p*−hydroxybenzaldehyde, and 4−nitrobenzaldehyde, to examine whether these probes show the reactivity to other aldehyde groups or not. In general, all of the probes (5 μM)) were treated with different analytes (500 μM) and FA (500 μM) for one hour, and then the fluorescence spectrum was measured. As shown in Figures S8 and S9, although **FA−1** provided the ratiometric result, its reactivity and selectivity towards FA are less than satisfactory, especially for NO_2_^−^, which could form diazonium salt with the amine group in **FA−1** (Figure S8 in Supplementary Materials). For **FA−5**, there is also no acceptable result obtained because of its low kinetic and reactivity (Figures S16 and S17 in Supplementary Materials). In the reactions of **FA−3** (Figures S12 and S13 in Supplementary Materials) and **FA−4** (Figures S14 and S15 in Supplementary Materials) with FA, although the tolerable selectivity has been achieved, the fluorescence intensity increment was relatively low, which was about 12 and 15−folds. With regards to **FA−2**, it exhibited the best selectivity for FA and other competitive species, which was more than 200−fold (Figure 2, Figures S10 and S11 in Supplementary Materials). Taking these results together, **FA−2** demonstrated the outstanding performance in terms of reaction kinetic and selectivity upon the reaction with FA, suggesting that **FA−2** could serve as an excellent candidate in cell imaging.

**Figure 3:**
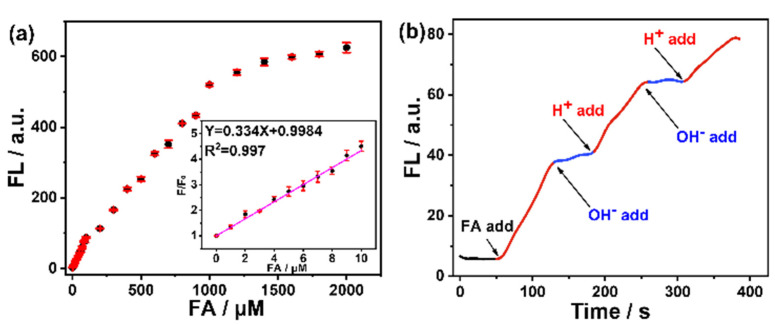
(**a**) Fluorescence titration of **FA−2** toward different concentrations of FA (0–2000 μM) at 530 nm in PBS buffer (pH 5, 10 mM). Inset: the relationship between the fluorescence intensity and the concentration of FA in the range of 0–10 μM. (**b**) Real−time monitoring of fluorescent intensity changes of **FA−2** (10 μM) toward FA (8 mM). The pH is switching between 5 and 7.4 by adding NaOH (1 mM) or HCl (1 mM) respectively. Ex: 380 nm, Em: 530 nm.

To evaluate the sensitivity of **FA−2** towards FA, the titration experiments were conducted in PBS buffer and monitored by fluorescence spectrum. As shown in Figure 3a and Figure S18, the free probe **FA−2** (5 μM) showed negligible fluorescence due to the PET effect in PBS buffer (pH 5, 10 mM), while the fluorescence was increased gradually upon the addition of FA. The fluorescence intensity at 530 nm matched well with added FA concentration from 0 to 1 mM, and the maximum intensity was reached when FA concentration was up to 2 mM. Specifically, FA−2 also displayed a good linear relationship (R^2^ = 0.997) with 0.65 μM of the limit of detection of and 2.56 μM of the limit of quantification. However, for **FA−1**, **FA−3**, **FA−4**, **FA−5**, the detection limit was 28.64 μM, 15.58 μM, 3.75 μM, and 89.55 μM, respectively (Figures S19–S22 in Supplementary Materials). FA−2 also displayed good recovery efficiency with different FA concentration using the standard curves (Table S1 in Supplementary Materials). The above result suggested that **FA−2** is sensitive enough to detect low concentration of FA in aqueous solution.

To further validate the pH effect on **FA−2**, the fluorescence intensity at 530 nm was recorded when the pH value was switched between 5.0 and 7.4. As shown in Figure 3b, the fluorescence signal at 530 nm gradually increased at pH 5.0. The reaction kinetics, however, was almost stopped when the pH was adjusted to 7.4 in the same reaction solution. Upon adjusting the pH back to 5.0, the fluorescence continued to increase again. By converting pH three times, the same result was obtained. Based on these results, **FA−2** performed better in recognizing FA in acidic media than in neutral and basic media, indicating that **FA−2** or its modified probe can be used to monitor FA fluctuation in lysosomes in living cells since the pH of the lysosome ranges from 4.0 to 6.0.

**Figure 4:**
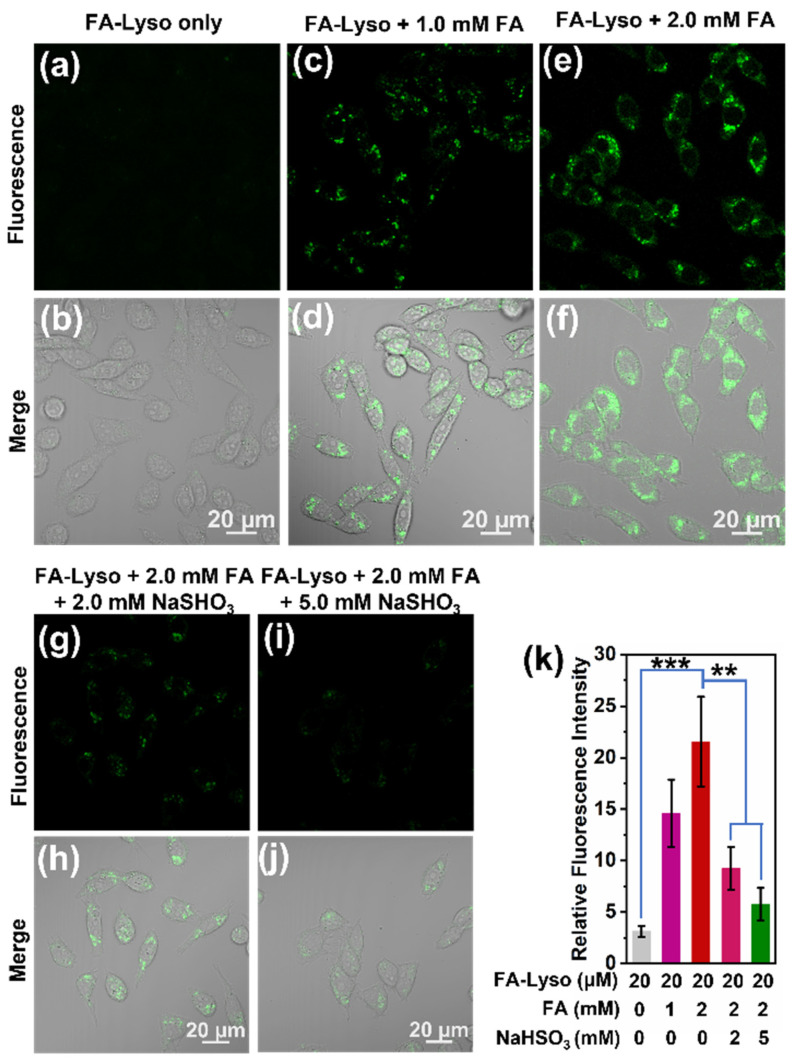
Confocal microscopy images of WI38 cells incubated with **FA−Lyso** and different concentrations of exogenous FA and NaHSO_3_. (**a**–**f**) Cells were incubated with **FA−Lyso** (20 μM) for 30 min and then treated with various concentrations of FA (0, 1, 2 mM) for 60 min. (**g**–**j**) Cells were incubated with **FA−Lyso** (20 μM) and NaSHO_3_ (2, 5 mM) for 30 min and then treated with FA (2 mM) for 60 min. (**k**) The relative fluorescence intensity of cells in Figure 4a–j. ** *p* < 0.01 *** *p* < 0.001 determined by Student’s *t* test.

Having established that **FA−2** can sense FA in vitro, we next evaluated its ability to monitor FA concentration in living cells. Firstly, we modified probe **FA−2** to **FA−Lyso**, in which we rationally introduce a morpholine group for lysosomal targeting purposes. After synthesis and structure characterization, the fluorescence response properties of FA detection were studied. The result indicates that **FA−Lyso** maintains fast kinetics and excellent sensitivity as **FA−2** (Figure S23 in Supplementary Materials). On the basis of the above results, the ability of **FA−Lyso** to monitor FA in living cells was assessed. Firstly, cytotoxicity of **FA−Lyso** was evaluated after 24 h of incubation with cells, and no obvious cytotoxicity was observed (Figure S24 in Supplementary Materials). To evaluate its capability of monitoring FA, different concentrations of FA (0, 1.0, 2.0 mM) were incubated with **FA−Lyso** in living cells for one hour, and the fluorescence was imaged by confocal microscopy. As shown in Figure 4, the cells incubated with **FA−Lyso** only displayed negligible fluorescence. In contrast, significant fluorescence signals can be observed in the cells containing **FA−Lyso** and exogenous FA. In addition, the fluorescence intensity enhanced gradually along with FA concentration increase (Figure 4c,e), demonstrating that **FA−Lyso** was capable of imaging exogenous FA in living cells.

To further validate the fluorescence signal was generated from the specific reaction between **FA−Lyso** and FA, we performed inhibition experiment with NaHSO_3_, which could act as an excellent scavenger through reacting with FA at the central carbonyl group [20,34]. The fluorescence signal in WI−38 cells, which were treated with **FA−Lyso** with FA showed a statistically significant difference. In the cells pretreated with NaHSO_3_, however, the fluorescence intensity obviously decreased. In addition, the fluorescence intensity decreased accordingly with the increasing NaHSO_3_ concentration, proving the capability of **FA−Lyso** to monitor FA fluctuation in living cells (Figure 4g–k).

**Figure 5:**
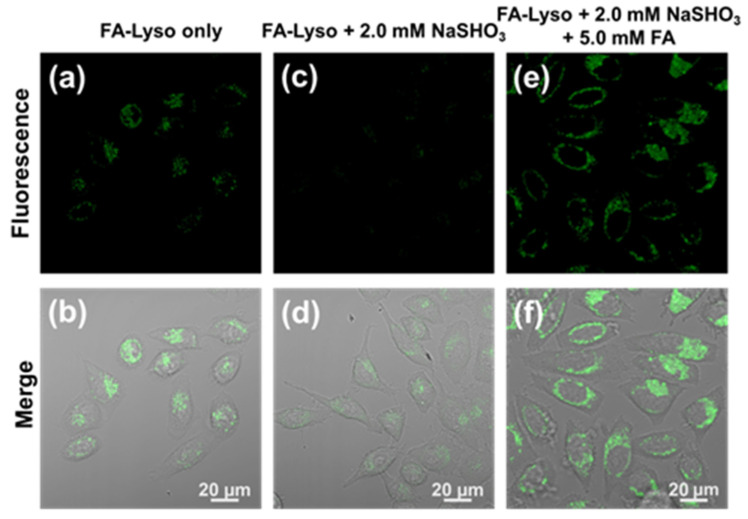
Confocal microscopy images of HeLa cells treated with **FA−Lyso** to detect endogenous FA. (**a**,**b**) Cells were incubated with **FA−Lyso** (20 μM) for 60 min. (**c**,**d**) Cells were incubated with NaSHO_3_ (2 mM) and **FA−Lyso** (20 μM) for 60 min. (**e**,**f**) Cells were incubated with NaSHO_3_ (2 mM), FA (5 mM), and **FA−Lyso** (20 μM) for 60 min.

Encouraged by the above results, we further evaluated the capability of **FA−Lyso** to detect endogenous FA in living cells. We chose HeLa cells for our experiments as this cell line has been reported to overexpress FA [35,36]. First, with the addition of **FA−Lyso** alone, significantly enhanced fluorescence was observed (Figure 5a,b). However, when cells were pretreated with NaHSO_3_ and then incubated with **FA−Lyso**, the fluorescence intensity was dramatically decreased, indicating that FA in living cells has been depleted (Figure 5c,d). Subsequently, cells were sequentially preincubated with NaHSO_3_, FA, and **FA−Lyso**, the recovered fluorescence signal can be observed again (Figure 5e,f). Collectively, these studies demonstrate that **FA−Lyso** is capable of detecting endogenous FA fluctuation in living cells.

Subsequently, we further evaluated whether the targeted organelle of **FA−Lyso** is lysosomes. WI−38 cells were incubated with **FA−Lyso** and commercially available dye Lyso−Tracker Red (lysosome dye) or Mito−Tracker Deep Red (mitochondrial dye) in the presence of FA for 1 h. As shown in Figure S25, bright yellow fluorescence was observed in the merged image of WI38 cells treated with **FA−Lyso** and Lyso−Tracker Red (Figure S25c in Supplementary Materials). In contrast, cells incubated with **FA−Lyso** and Mito−Tracker Deep Red did not show significant overlap in green and red color regions (Figure S25h in Supplementary Materials). Meanwhile, the fluorescence intensity profiles of the regions of interest displayed synchronous changes in the presence of **FA−Lyso** and Lyso−Tracker Red (Figure S25i in Supplementary Materials). In contrast, in the case of Mito−Tracker Deep Red, no obvious synchronous changes were observed (Figure S25j in Supplementary Materials). Taken together, these results proved that **FA−Lyso** can be used to monitor exogenous and endogenous FA levels in lysosomes in living cells.

## 4. Conclusions

In summary, using 2−aza−Cope rearrangement strategy, five fluorescent probes, **FA−1**, **FA−2**, **FA−3**, **FA−4**, and **FA−5**, were designed from the same fluorophore to systemically screen their sensing ability of FA in aqueous buffer and living cells. Experiment results demonstrated that among all five probes, **FA−2** achieved improved kinetics, selectivity, and sensitivity under weak acid condition. Furthermore, the probe **FA−Lyso**, derived from **FA−2**, exhibited excellent ability to visualize exogenous and endogenous FA fluctuations in lysosomes in living cells. Taken together, this study offers an attractive design strategy for FA probes based on 2−aza−Cope rearrangement. Moreover, this design strategy has been applied successfully for FA detection in living cells, demonstrating its robust utility.

## Data Availability

Not applicable.

## Supplementary Materials

The following Supplementary Materials can be downloaded at: https://www.mdpi.com/article/10.3390/bios12100855/s1.
