# Supplementary material for: Systematic Screening of Trigger Moieties for Designing Formaldehyde Fluorescent Probes and Application in Live Cell Imaging
Source: Biosensors (Basel). 2022 Oct 10;12(10):855. doi: 10.3390/bios12100855 (PMC9599387; doi:10.3390/bios12100855)
Supplement: Supplementary file 1 [file biosensors-12-00855-s001.zip › biosensors-1904615-supplementary.pdf]

Supplemental Schemes, Figures and Tables

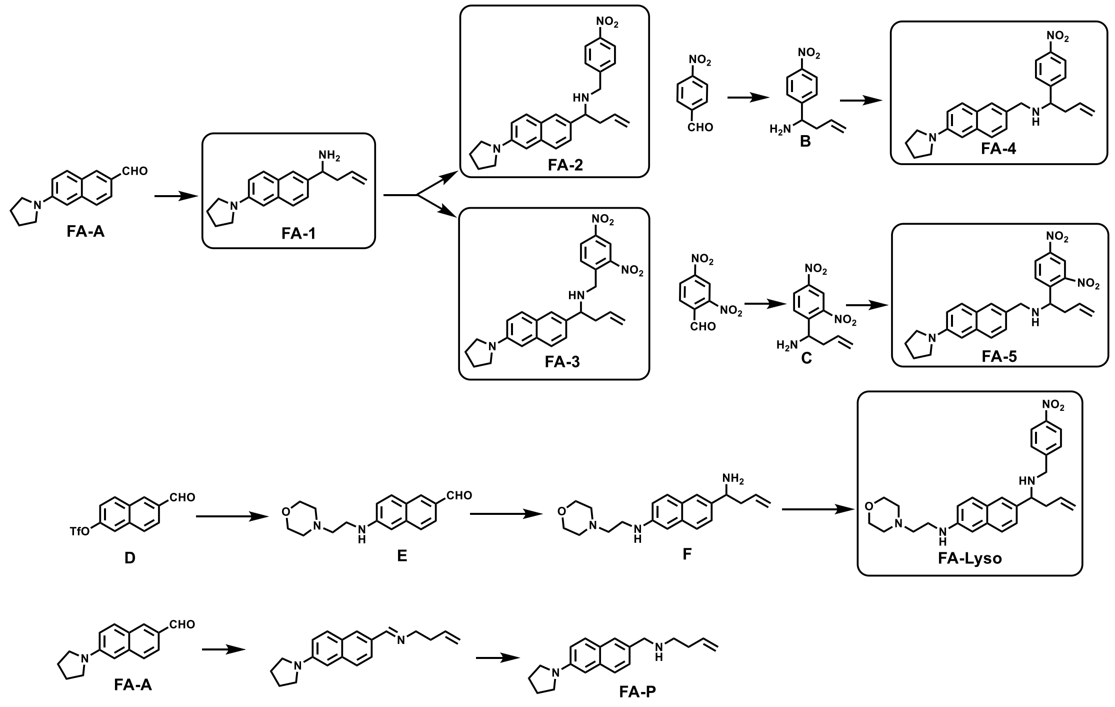

Scheme S1. The synthetic route of FA fluorescent probes (**FA-1**, **FA-2**, **FA-3**, **FA-4**, **FA-5** and **FA-Lyso**).

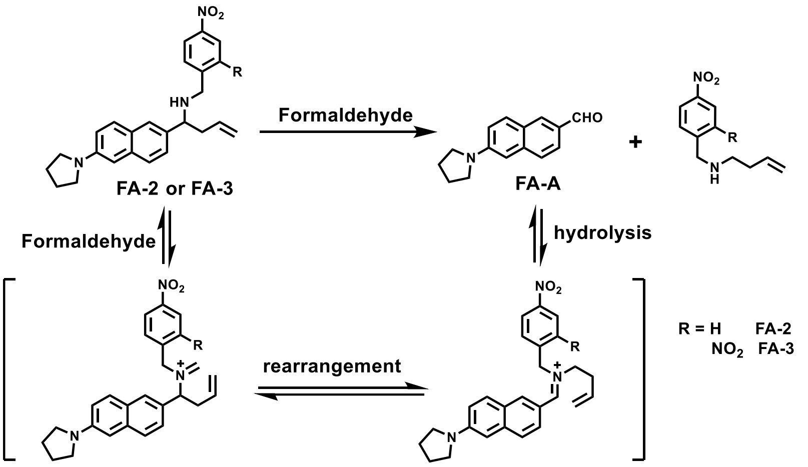

Scheme S2. Reaction mechanism of **FA-2** or **FA-3** with FA.

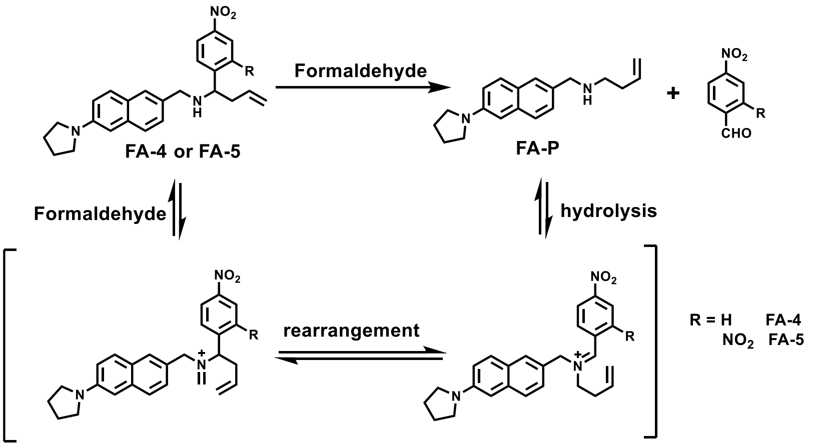

Scheme S3. Reaction mechanism of **FA-4** or **FA-5** with FA.

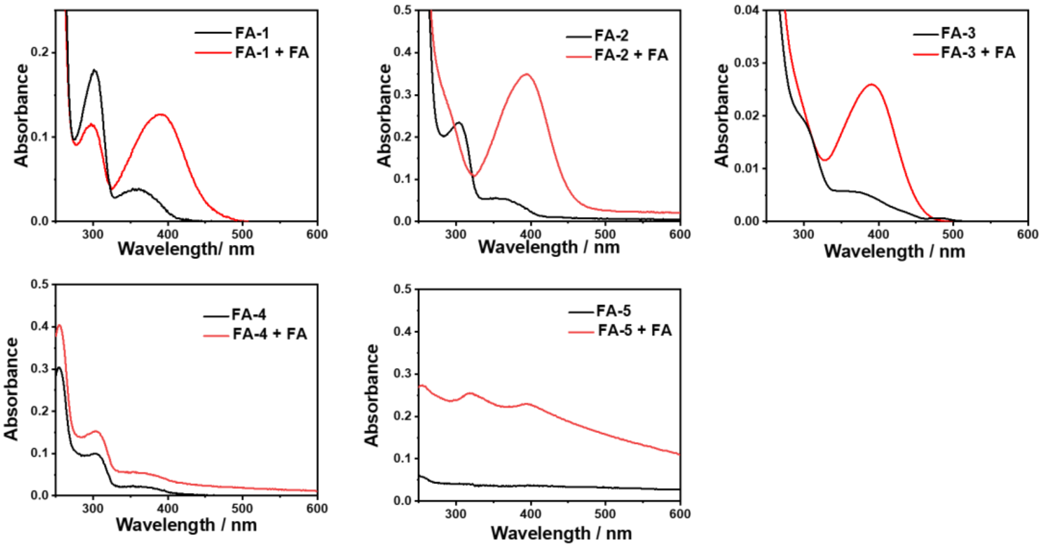

Figure S1. Absorption spectra of **FA-1~FA-5** (10 μM) in the absence and presence of FA (4 mM) in PBS buffer (pH 5, 10 mM).

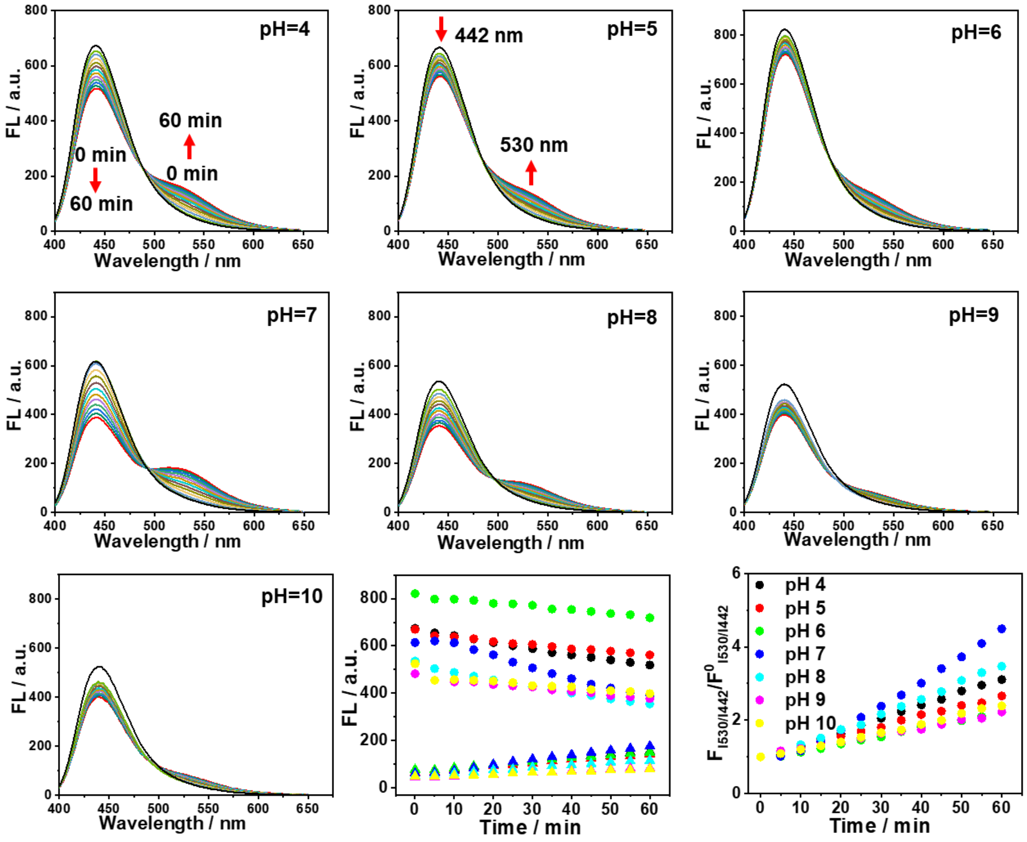

Figure S2. Time-dependent emission spectra of **FA-1** (5 μM) and FA (2 mM) in PBS buffer with different pH value (4–10). Ex: 380 nm.

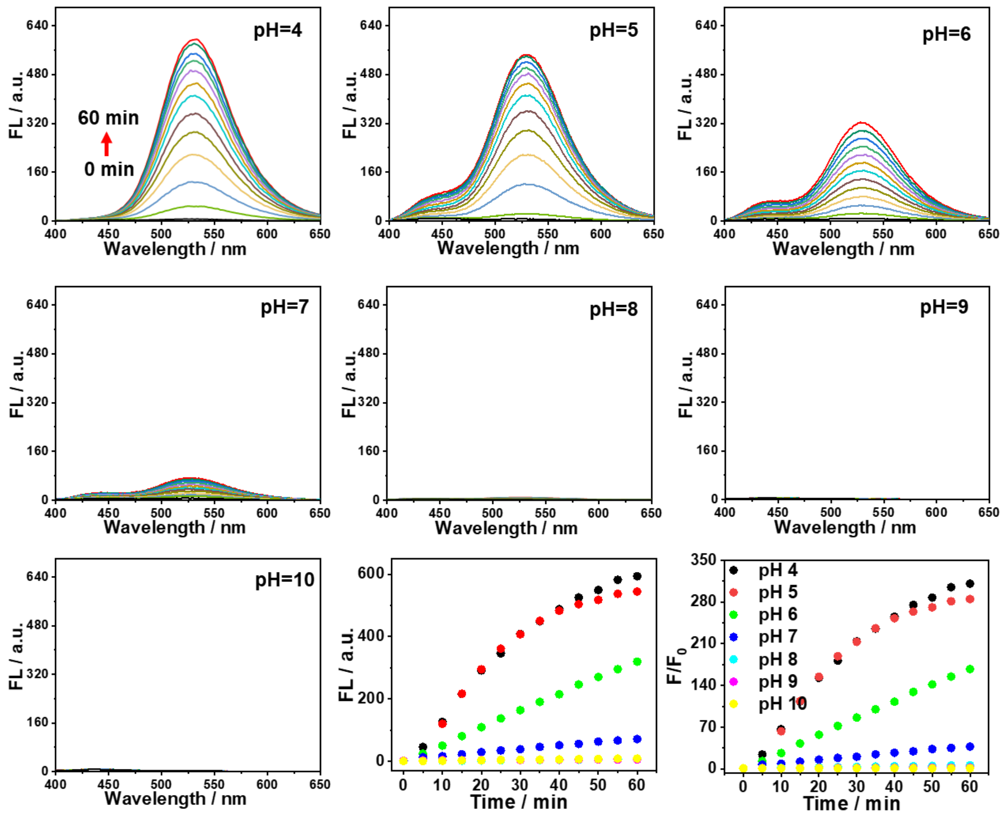

Figure S3. T Time-dependent emission spectra of **FA-2** (5 μM) and **FA** (2 mM) in PBS buffer with different pH value (4–10). Ex: 380 nm.

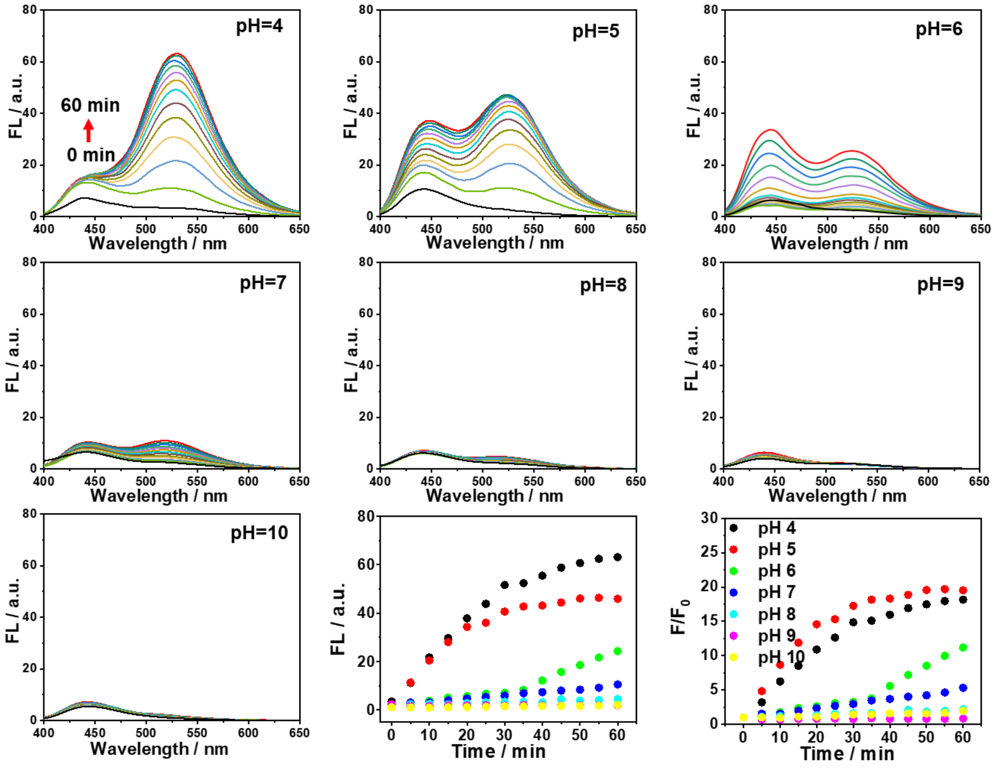

Figure S4. Time-dependent emission spectra of **FA-3** (5 μM) and FA (2 mM) in PBS buffer with different pH value (4–10). Ex: 380 nm.

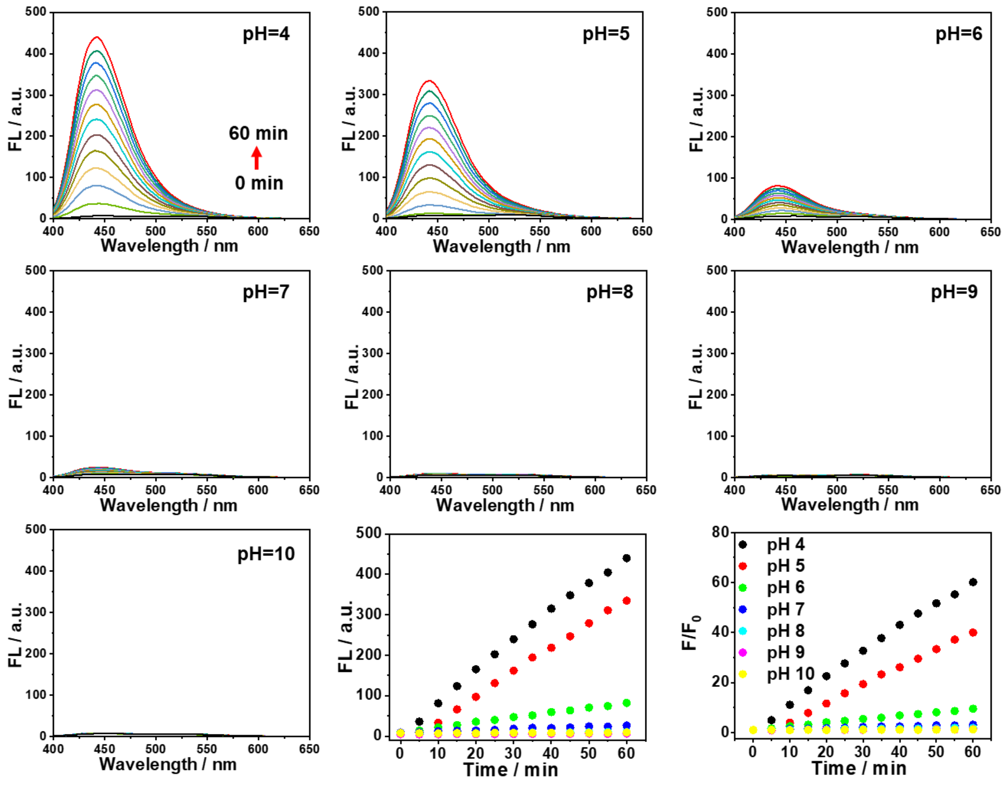

Figure S5. Time-dependent emission spectra of **FA-4** (5 μM) and FA (2 mM) in PBS buffer with different pH value (4–10). Ex: 365 nm.

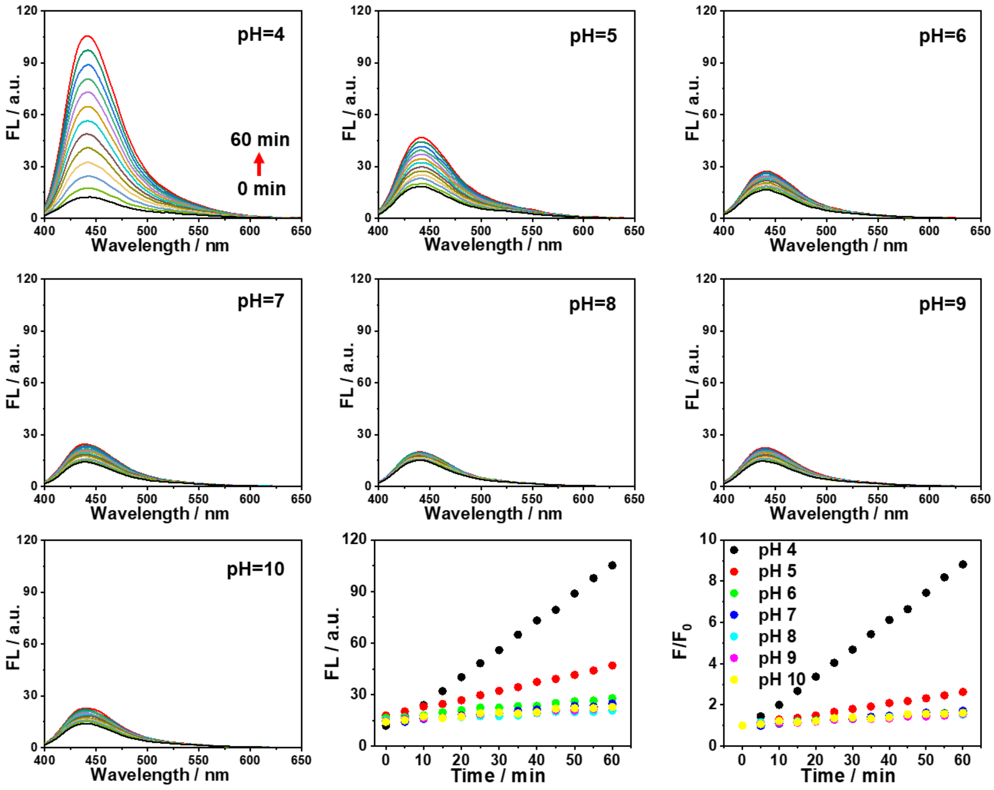

Figure S6. Time-dependent emission spectra of **FA-5** (5 μM) and FA (2 mM) in PBS buffer with different pH value (4-10). Ex: 365 nm.

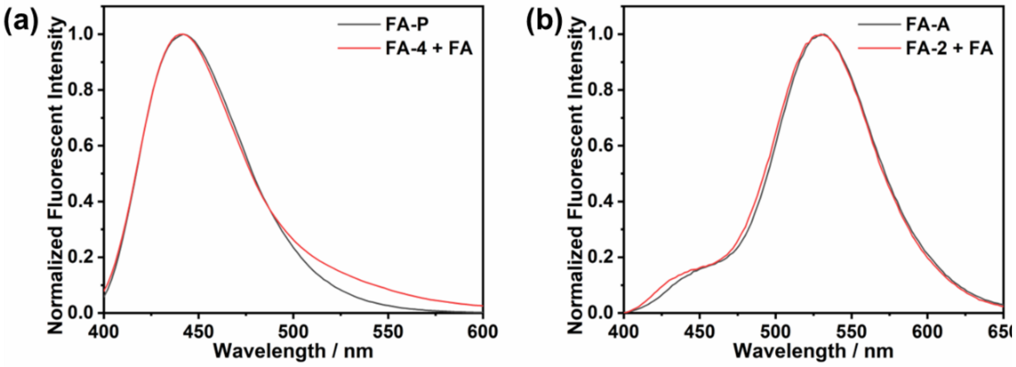

Figure S7. (**a**) Fluorescent stacking spectra of **FA-P** and the reaction of FA-4 with FA; (**b**) Fluorescent stacking spectra of **FA-A** and the reaction of FA-2 with FA.

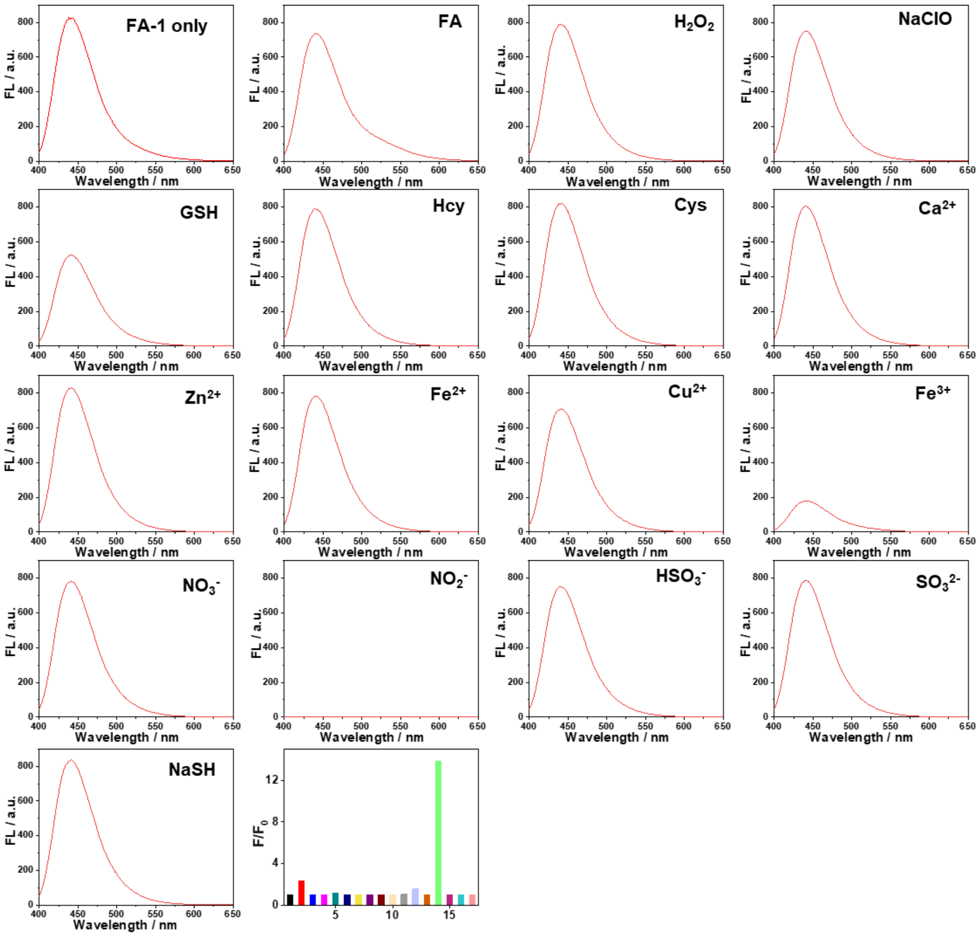

Figure S8. Selectivity test of **FA-1** (5 μM) towards FA (500 μM) and various relevant molecules and ions (500 μM). Ex: 380 nm.

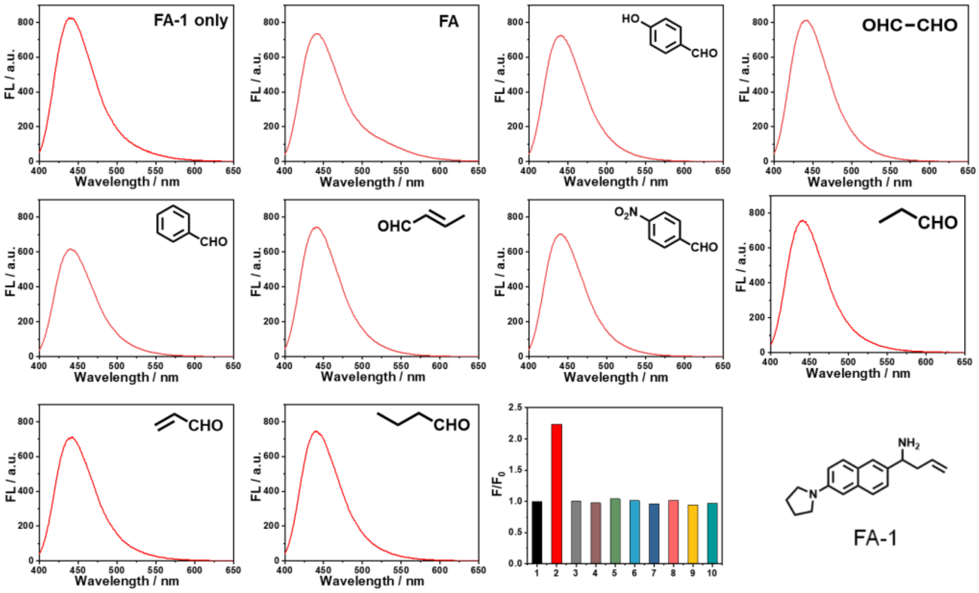

Figure S9. Selectivity test of **FA-1** (5 μM) towards FA (500 μM) and various compounds (500 μM) containing aldehyde group. Ex: 380 nm.

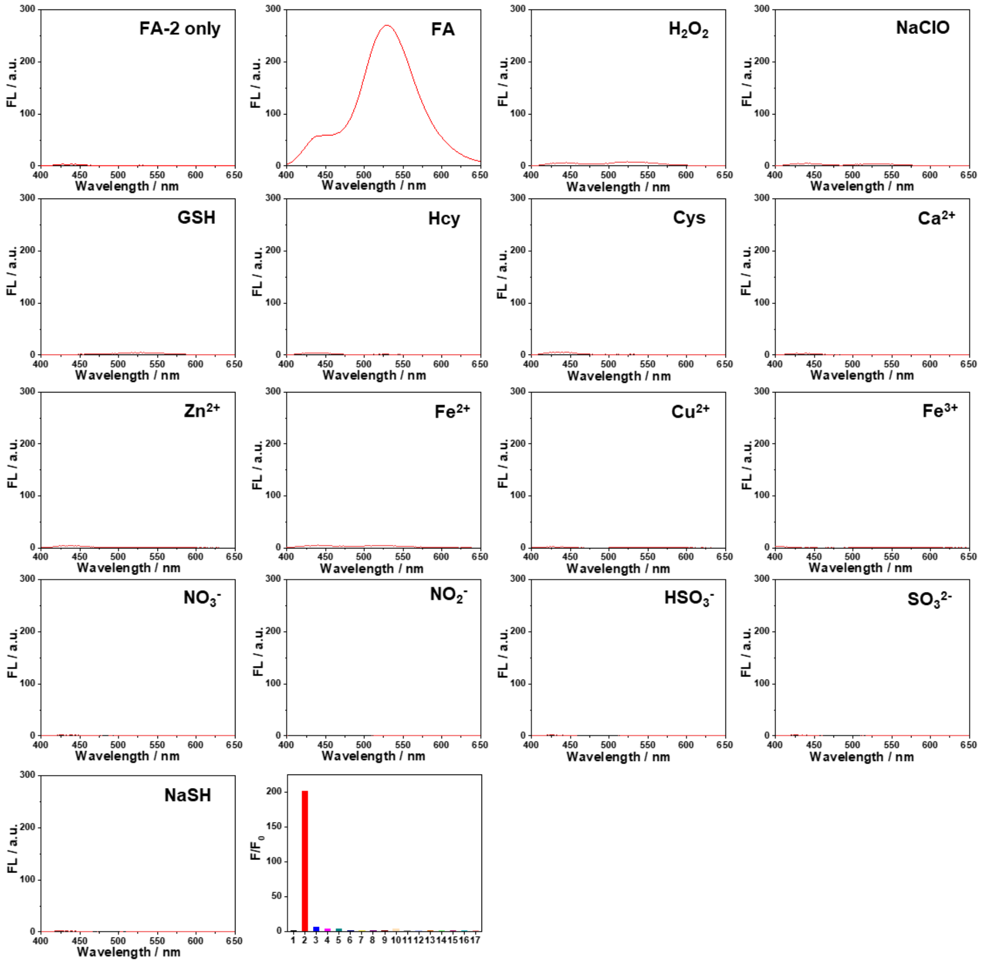

Figure S10. Selectivity test of **FA-2** (5 μM) towards FA (500 μM) and various relevant molecules and ions (500 μM). Ex: 380 nm.

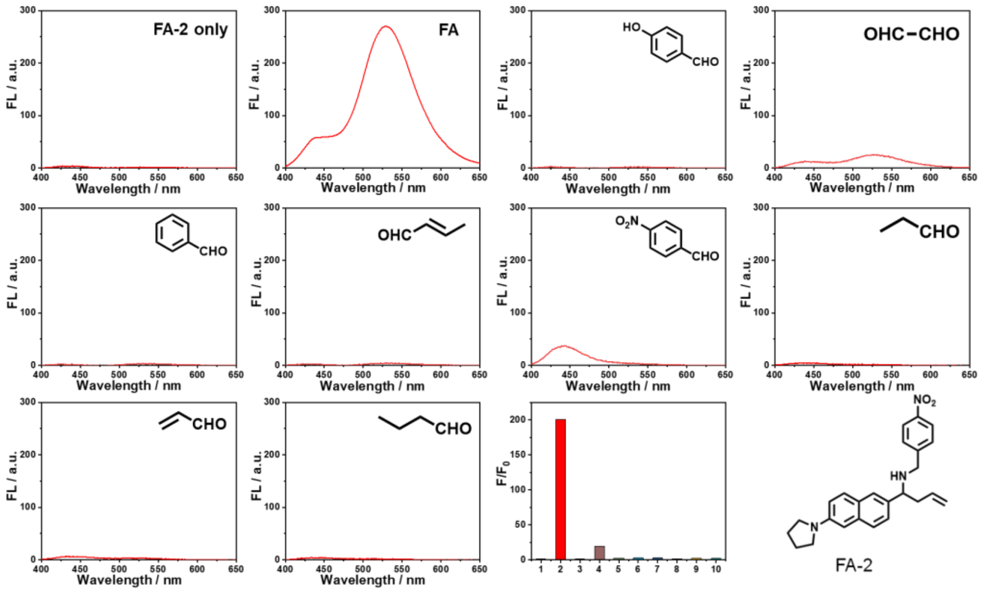

Figure S11. Selectivity test of **FA-2** (5 μM) towards FA (500 μM) and various compounds (500 μM) containing aldehyde group. Ex: 380 nm.

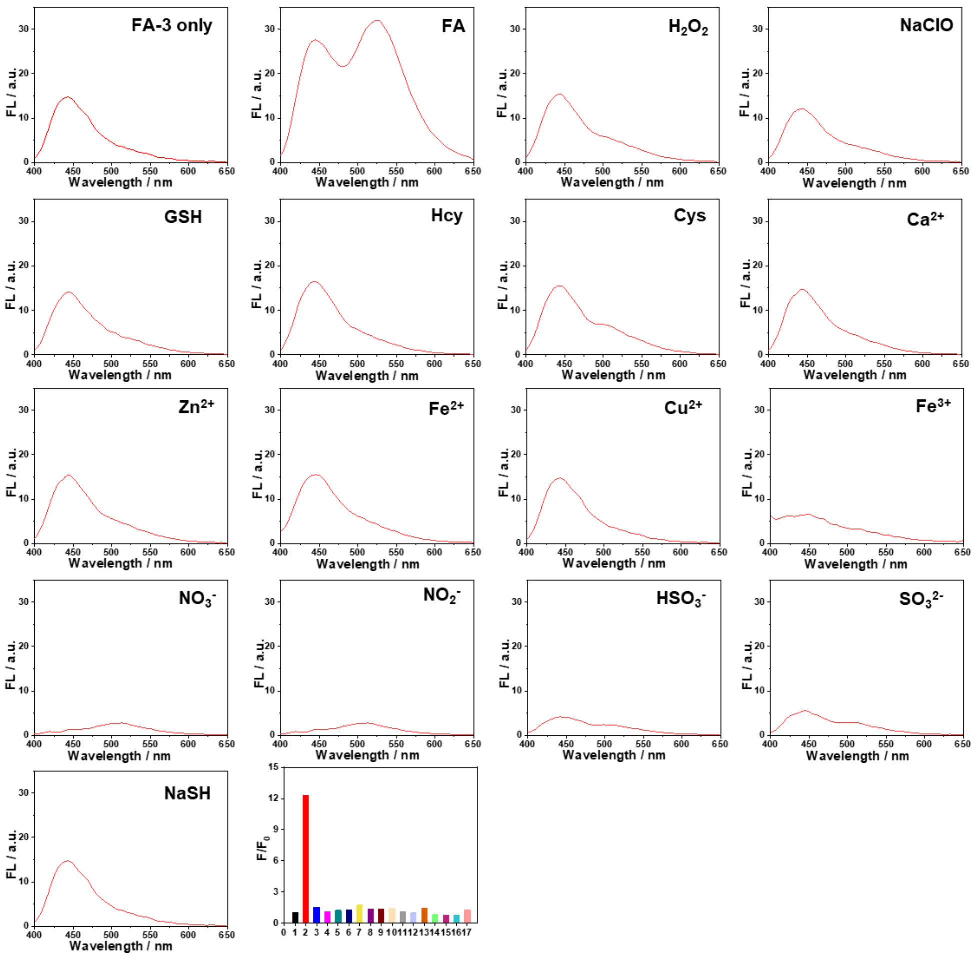

Figure S12. Selectivity test of **FA-3** (5 μM) towards FA (500 μM) and various relevant molecules and ions (500 μM). Ex: 380 nm.

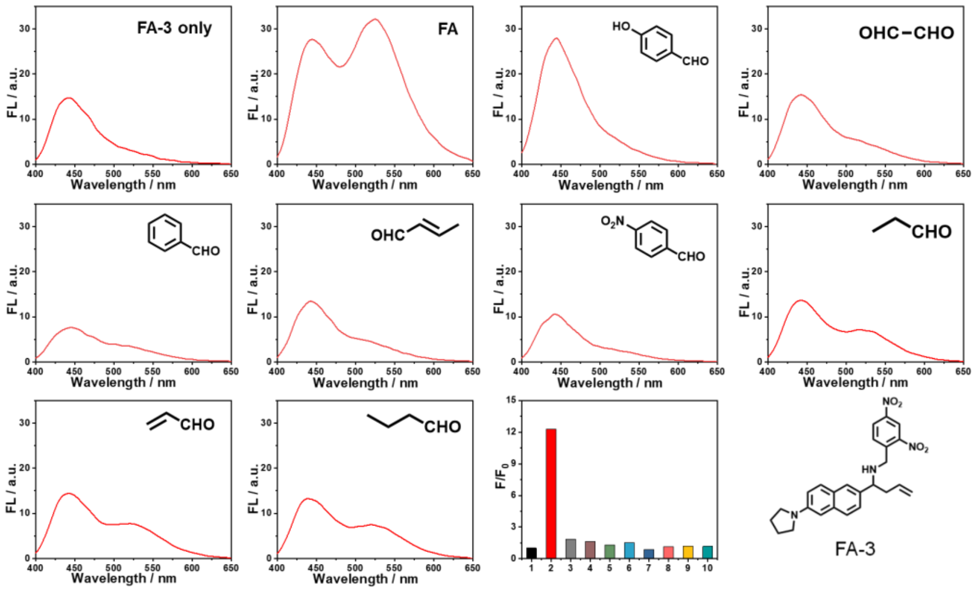

Figure S13. Selectivity test of **FA-3** (5 μM) towards FA (500 μM) and various compounds (500 μM) containing aldehyde group. Ex: 380 nm.

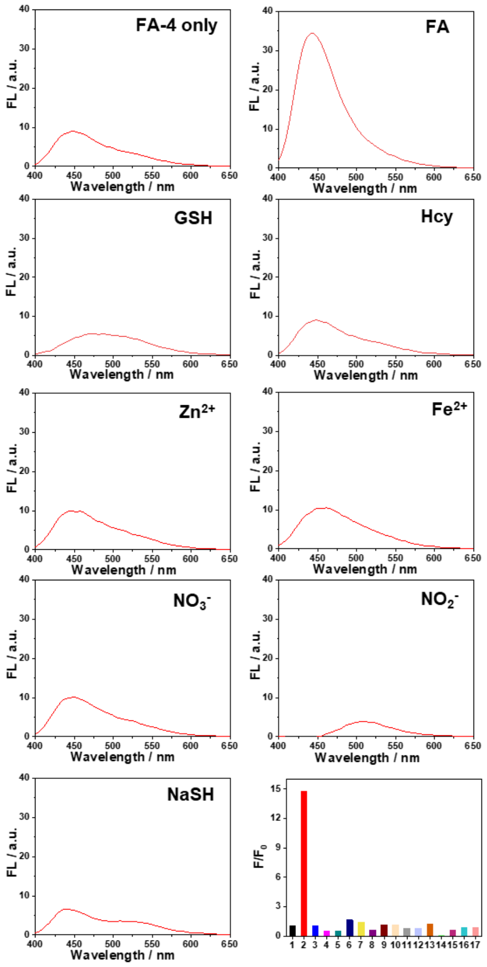

Figure S14. Selectivity test of **FA-4** (5 μM) towards FA (500 μM) and various relevant molecules and ions (500 μM). Ex: 365 nm.

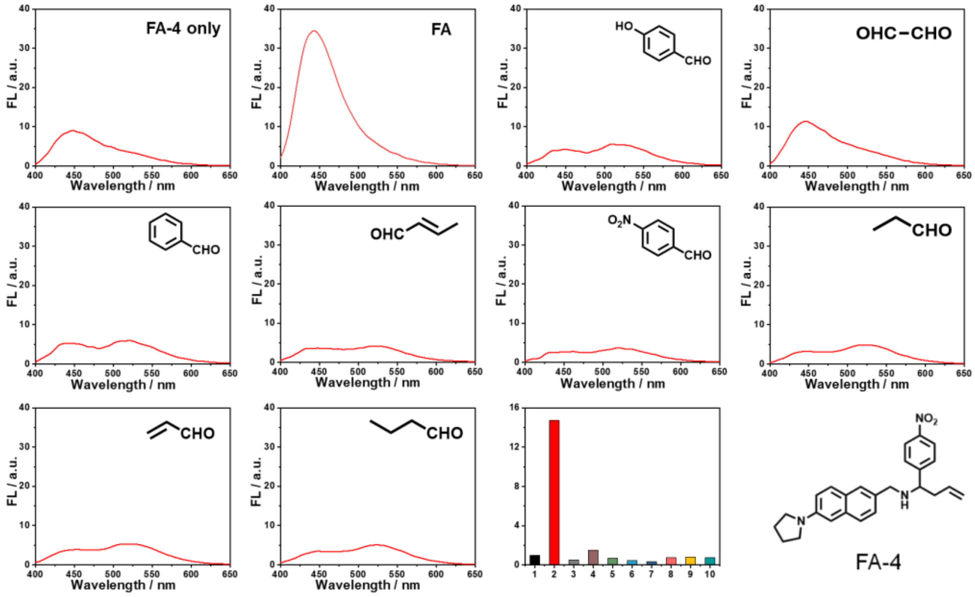

Figure S15. Selectivity test of **FA-4** (5 μM) towards FA (500 μM) and various compounds (500 μM) containing aldehyde group. Ex: 365 nm.

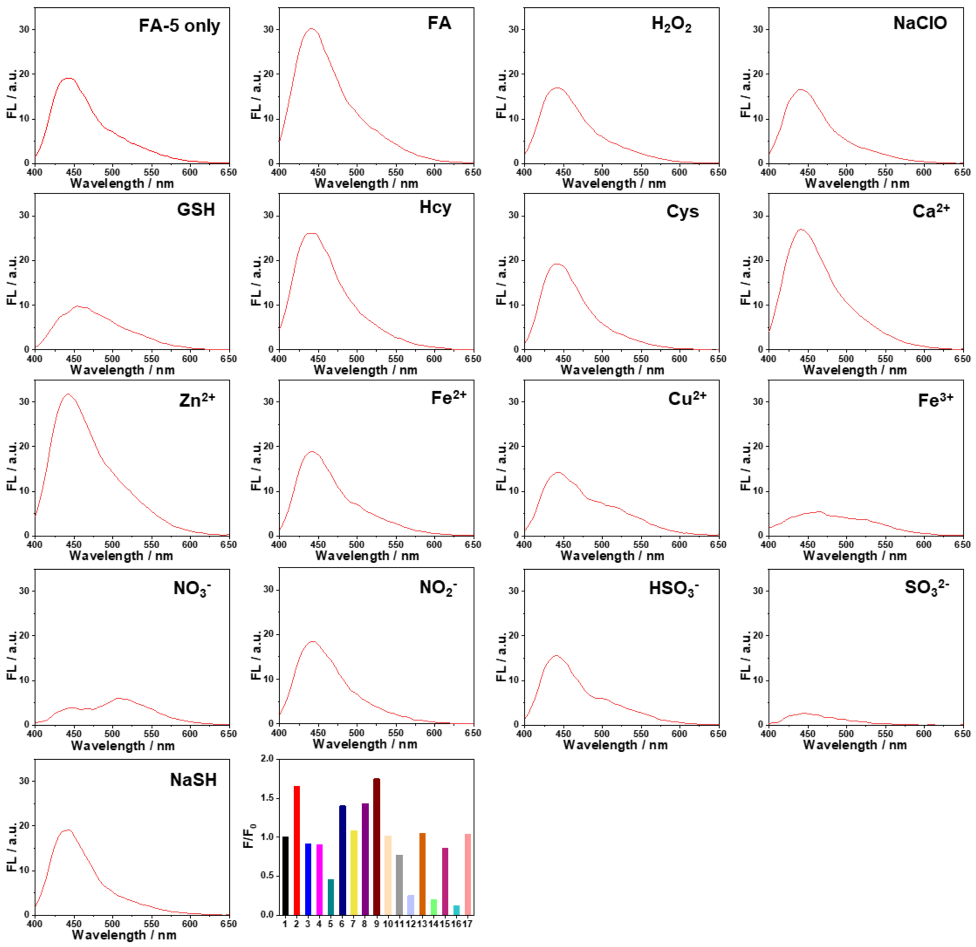

Figure S16. Selectivity test of **FA-5** (5 μM) towards FA (500 μM) and various relevant molecules and ions (500 μM). Ex: 365 nm.

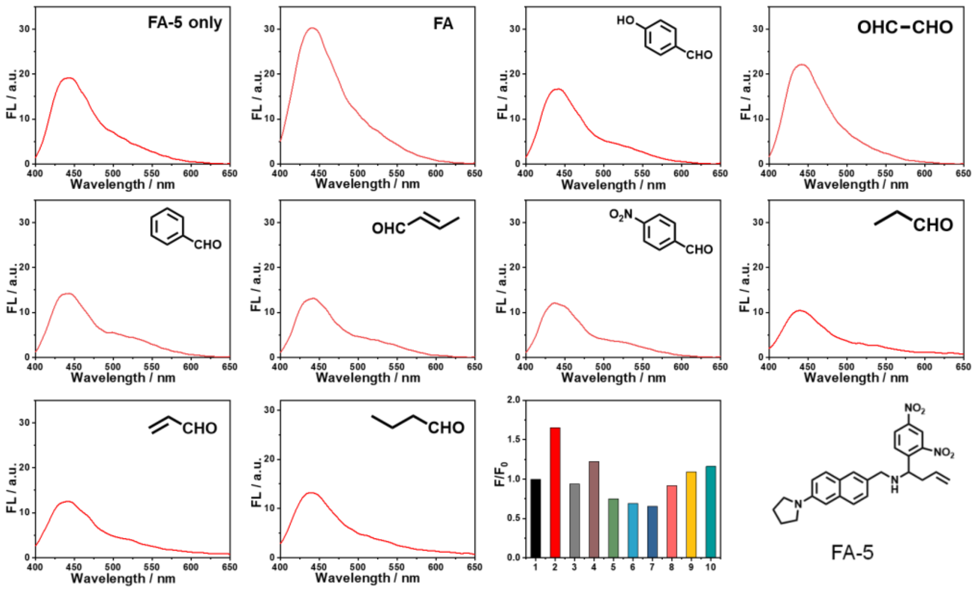

Figure S17. Selectivity test of **FA-5** (5 μM) towards FA (500 μM) and various compounds (500 μM) containing aldehyde group. Ex: 365 nm.

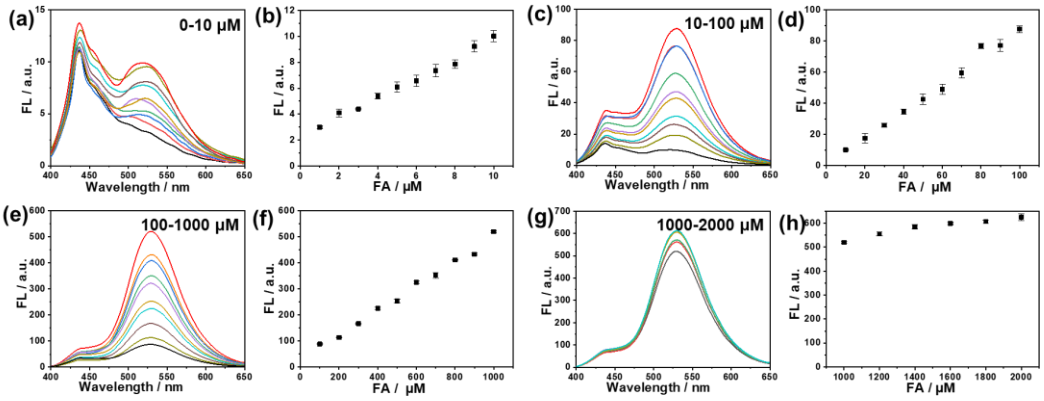

Figure S18. Fluorescence spectra and fluorescence intensity at 530 nm of **FA-2** (5 µM) reacting with different concentration FA. (**a**,**b**): 1–10 µM; (**c**,**d**) 10–100 µM; (**e**,**f**) (100–1000 µM); (**g**,**h**) (1000–2000 µM). Ex: 380 nm.

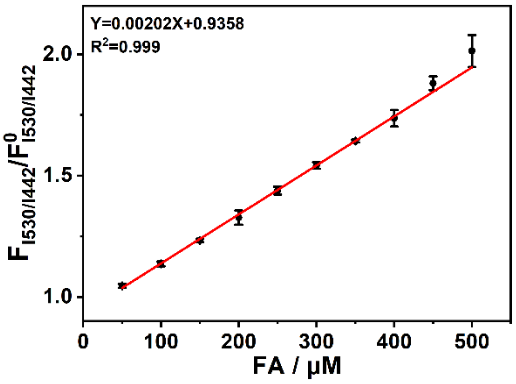

Figure S19. The relationship of **FA-1** between the fluorescence intensity increased fold and the concentration of FA in the range of 100-1000 µM.

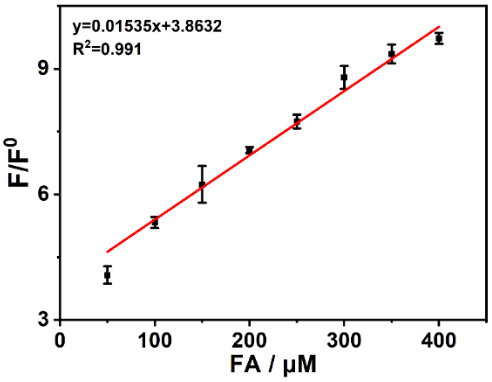

Figure S20. The relationship of **FA-3** between the fluorescence intensity increased fold and the concentration of FA in the range of 50-400 µM.

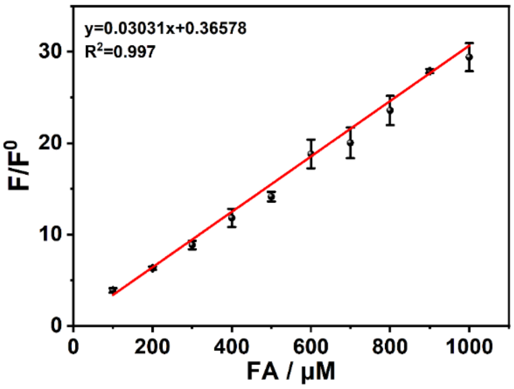

Figure S21. The relationship of **FA-4** between the fluorescence intensity increased fold and the concentration of FA in the range of 100-1000 μM.

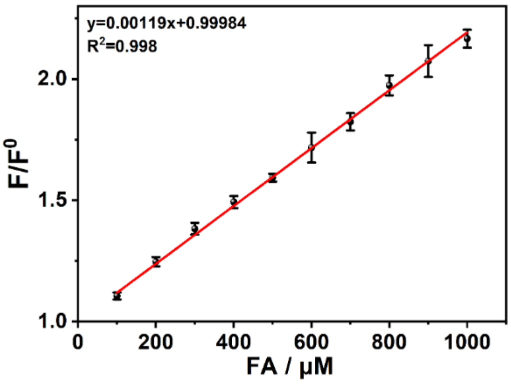

Figure S22. The relationship of **FA-5** between the fluorescence intensity increased fold and the concentration of FA in the range of 100-1000 μM.

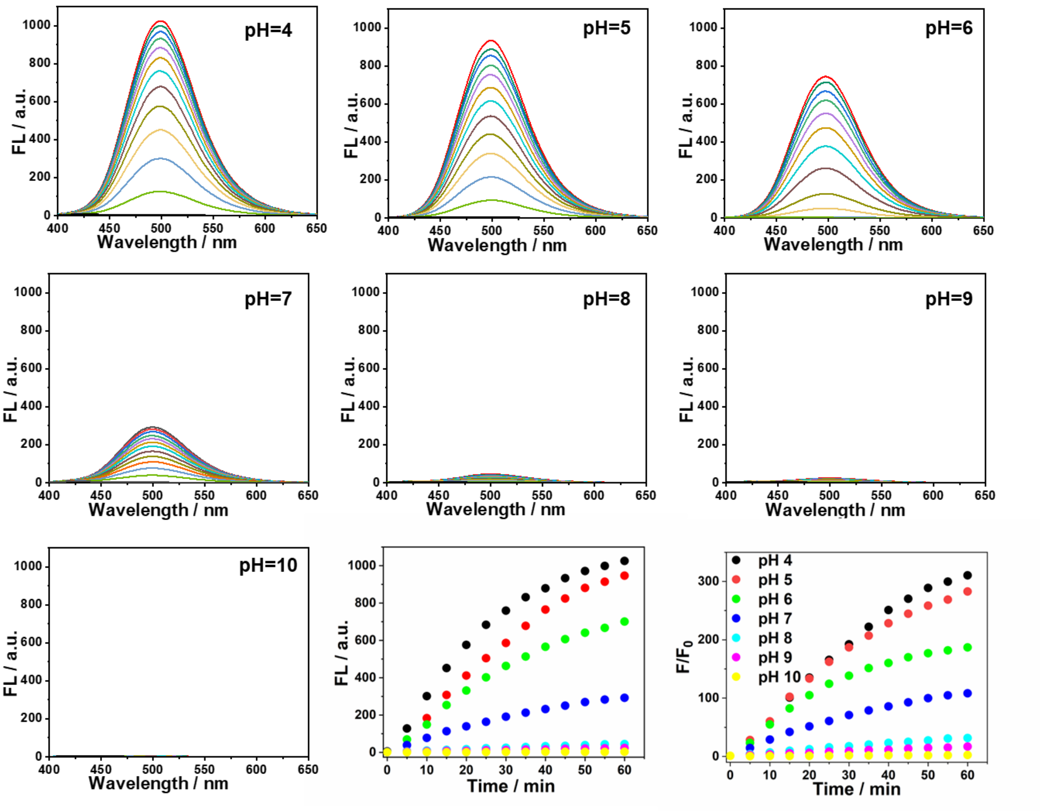

Figure S23. Time-dependent emission spectra of **FA-Lyso** (5 μM) and FA (2 mM) in PBS buffer with different pH value (4-10). Ex: 380 nm.

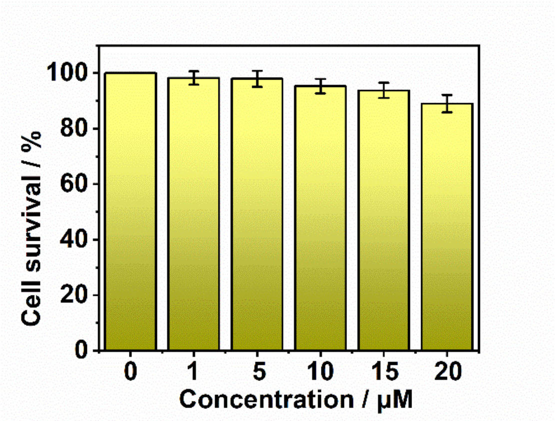

Figure S24. Cytotoxicity assays of **FA-Lyso** at different concentrations.

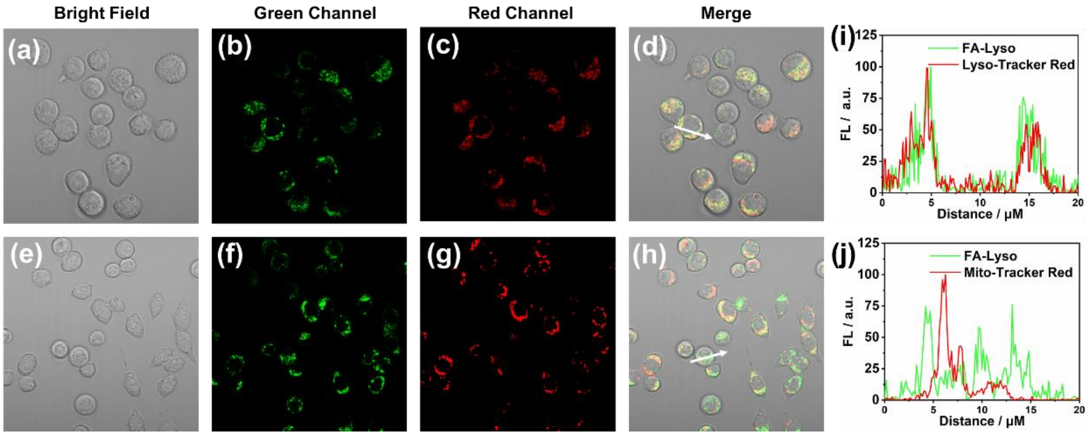

Figure S25. (**a**–**h**) Confocal fluorescence images of intracellular co-localization in WI38 cells. Cells were incubated with **FA-Lyso** (20 μM) for 30 min and treated with FA for another 120 min, then were stained with Lyso-Tracker Red and Mito-Tracker Deep Red (second line), respectively. (**a**,**e**) bright filed; (**b**,**f**) green channel; (**c**,**g**) red channel; (**d**,**h**) merge image of b and c or e and f; (**i**,**j**) fluorescence intensity profile of the area of interest across WI38 cells in Figure 25d and Figure 25h.

Table S1. Repeatability test of FA-2.

FA contents /μM	Test 1 / μM	Test 2 / μM	Test 3 / μM	Average / μM	Recovery efficiency
2	1.530	1.583	1.636	1.583	79.15%
4	4.029	3.592	3.937	3.853	96.23%
6	5.123	5.836	5.834	5.628	93.80%
8	7.496	7.819	7.927	7.747	96.84%

Table S2. Comparison of our probe and other fluorescent FA probes.

Probe	λ_{ex}/nm	λ_{em}/nm	FEF/fold[a]	Limit of detection	Detection environments	Reaction time	Imaging application
P-FA ACS Appl. Bio Mater., 2019, 2, 555	420	452↓ 480↓ 550↑	15	6.1 μM	DMSO	90 min	HeLa cell
TPE-FA ACS Sens., 2018, 3, 2112	335	480	4	0.036 mg/m³	DMSO/H_2O (V/V = 1/9)	37 °C, 180 min	none
RFFP Chem. Commun., 2016, 52, 4029	318	359↓ 451↑	53.2 (pH=7.4)	59.6 μM (pH=7.4) 18.7 μM (pH=4.5)	PBS (25 mM, containing 1% acetone)	200 min	HeLa cell
Naph-FA Anal. Methods, 2018, 10, 2963	395	518	11.2	0.22 μM	PBS (25 mM, pH = 7.4, containing 20% DMSO)	180 min	HeLa cell
PIPBA Chem. Sci., 2017, 8, 7851	350	440↓ 520↑	92.2	0.84 μM	DMSO/PBS (50/50, v/v, pH = 7.4, 20 mM)	120 min	HeLa cell, mouse renal tissue, Zebrafish
B1 Anal. Chim. Acta, 2018, 1033, 180-	390	472	not mentioned	0.107 μM	EtOH/HEPES (10 mM, pH = 7.4, 1:99, v/v)	not mentioned	HEK293T cells
RFAP-2 Chem. Sci., 2017, 8, 4073	420↓ 470↑	510	6	0.3 μM	PBS (20 mM, pH = 7.4)	37 °C, 120 min	HEK293T cells
Lyso-TPFP Chem. Commun., 2017, 53, 6520	405	506↑ 566↓	not mentioned	3 μM	PBS (50 mM, pH = 5)	not mentioned	HepG2 cells, HeLa cells, tissues in the abdomen of mice
FAP-1 J. Am. Chem. Soc., 2015, 137, 10886	645	662	45	5 μM	PBS (20 mM, pH = 7.4)	37 °C, 120 min	HEK293T cells, MCF-7 cells
BD-CHO Front. Chem., 2018, 6, 488	460	578	55	9.7 μM	HEPES/DMSO (V/V = 1:1, 20 mM, pH = 7.4)	180 min	HepG2 cells, MCF-7 cells, kidney tissues, Daphnia magna
FA-2 This work	380	530	280	0.65 μM	PBS (10 mM, pH = 5)	60 min	WI38 cells HeLa cells

[a] The fluorescence intensity changes before and after incubation with FA.

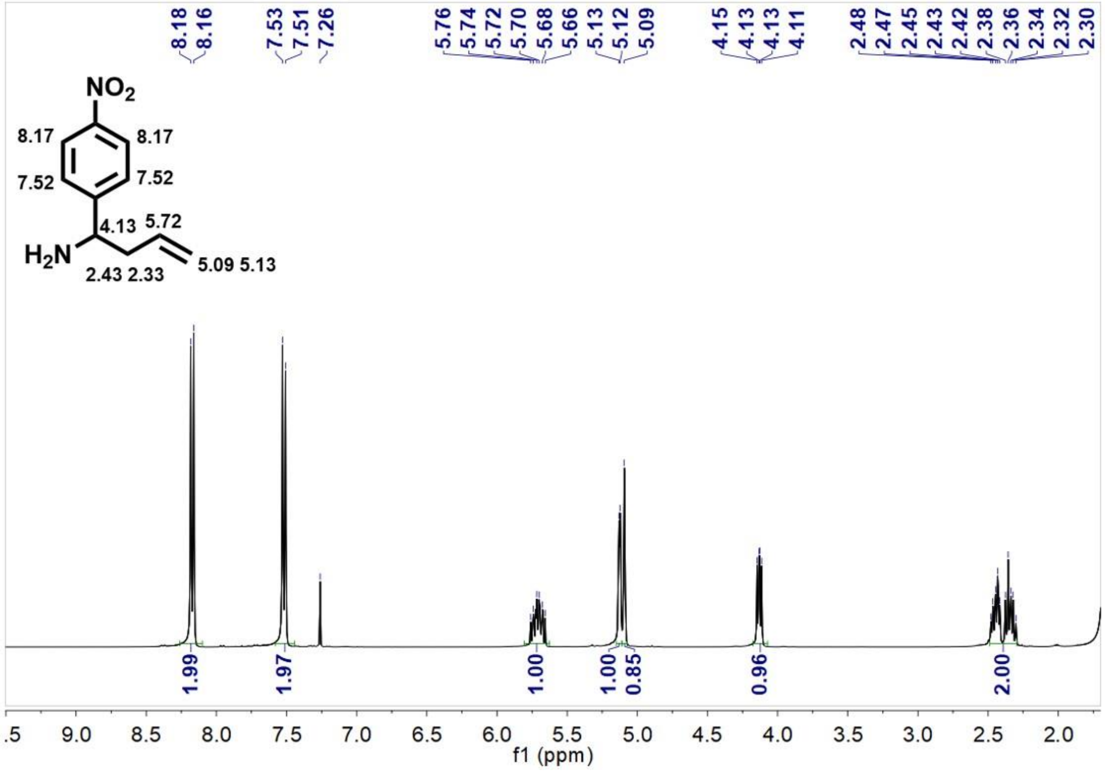

Figure S26. ^{1}H NMR spectrum of compound B ($CDCl_3$).

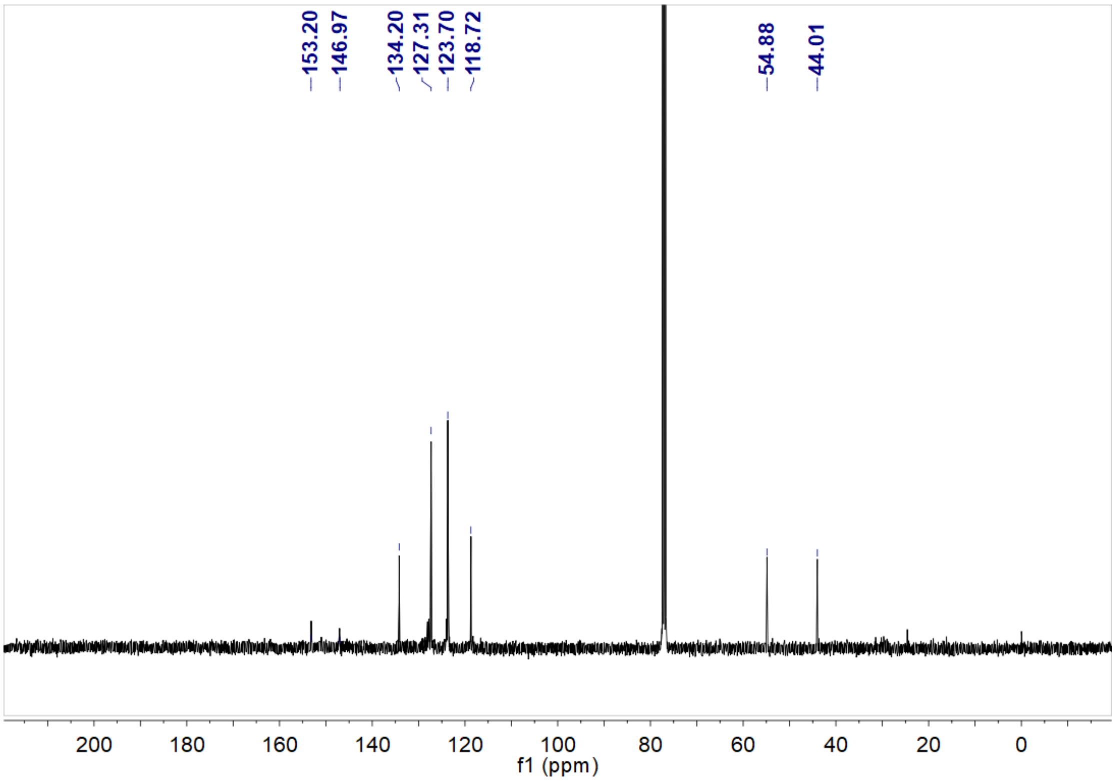

Figure S27. ^{13}C NMR spectrum of compound B ($CDCl_3$).

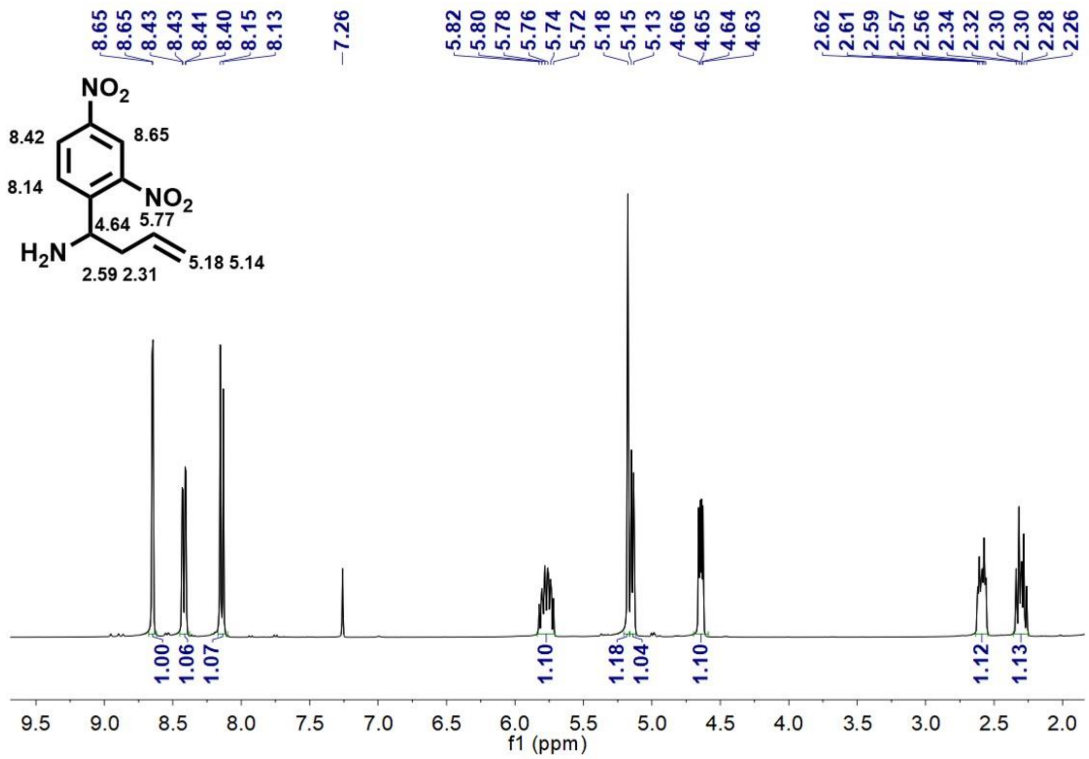

Figure S28. ^{1}H NMR spectrum of compound C(CDCl3).

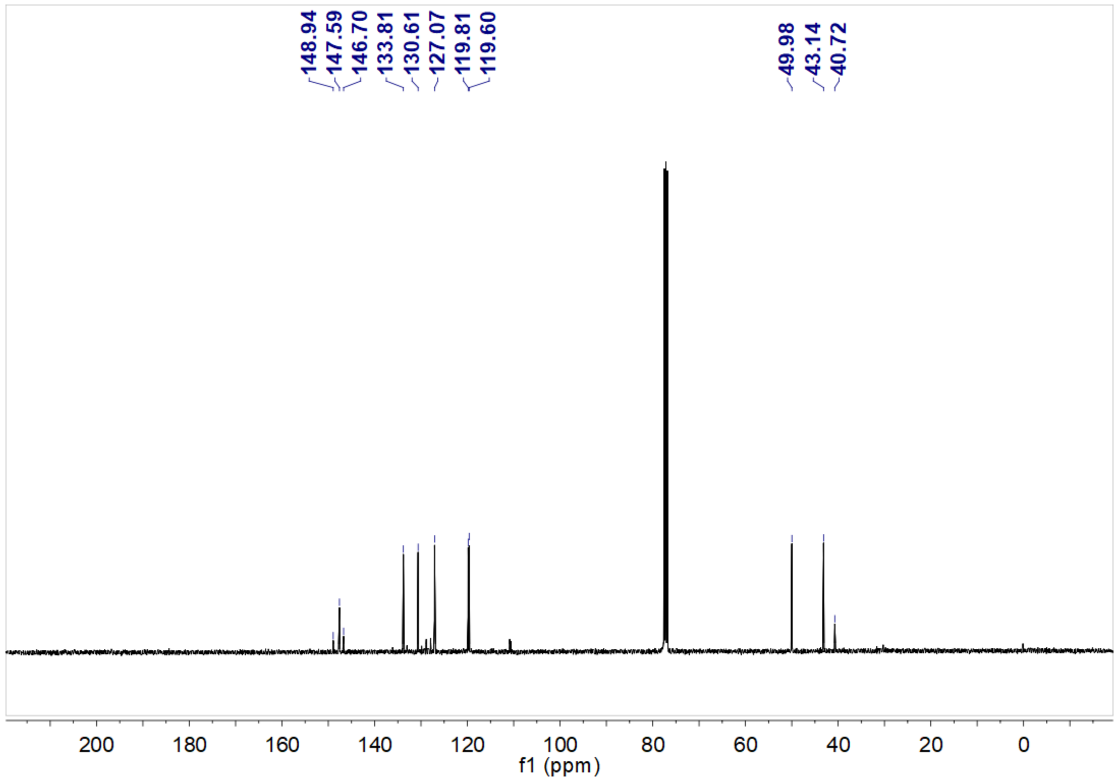

Figure S29. ^{13}C NMR spectrum of compound C (CDCl3).

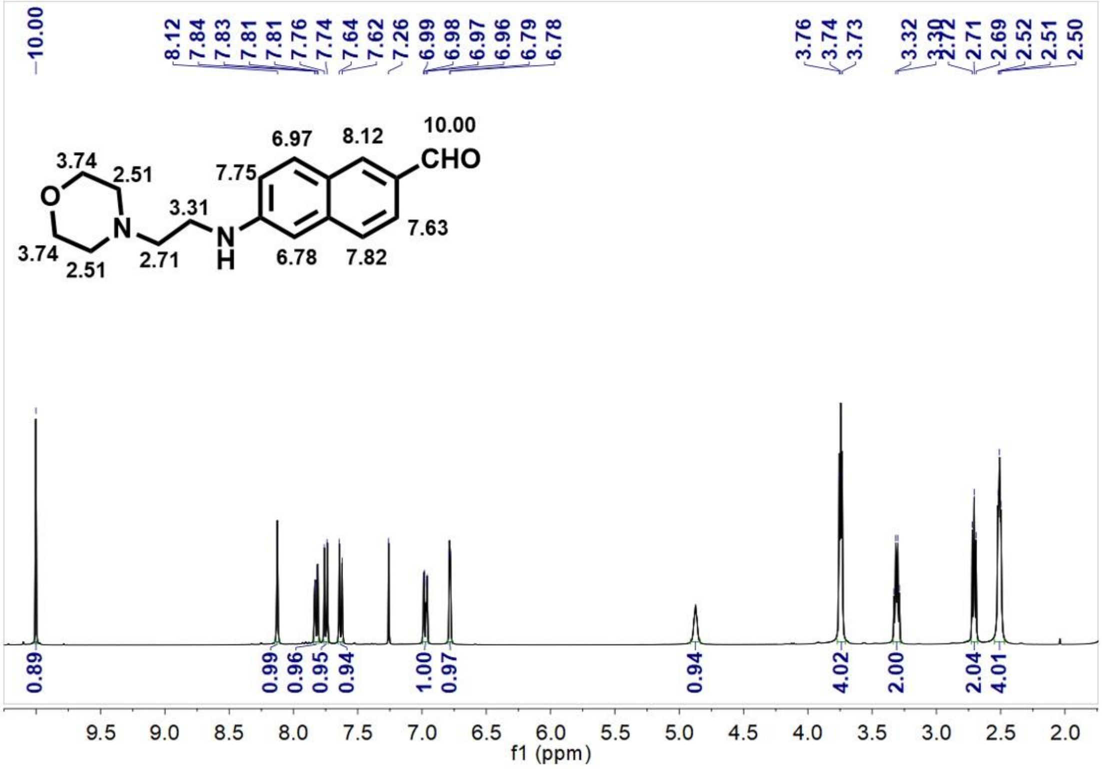

Figure S30. ^{1}H NMR spectrum of compound E ($CDCl_3$).

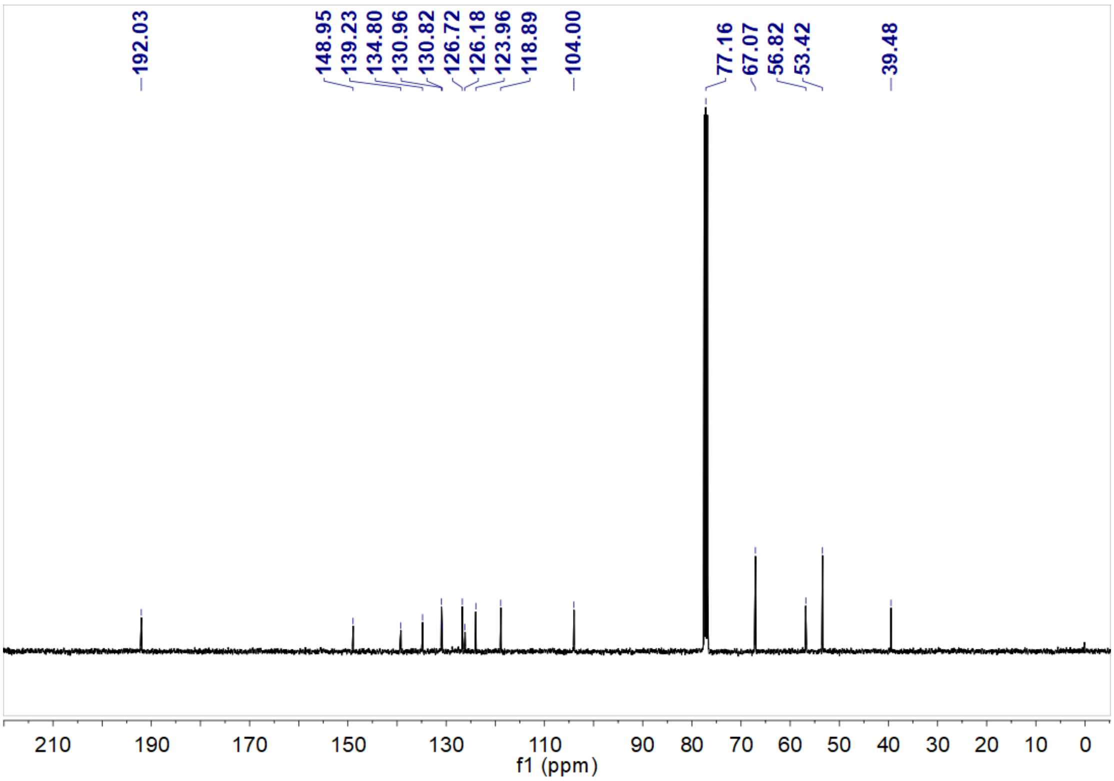

Figure S31. ^{13}C NMR spectrum of compound E ($CDCl_3$).

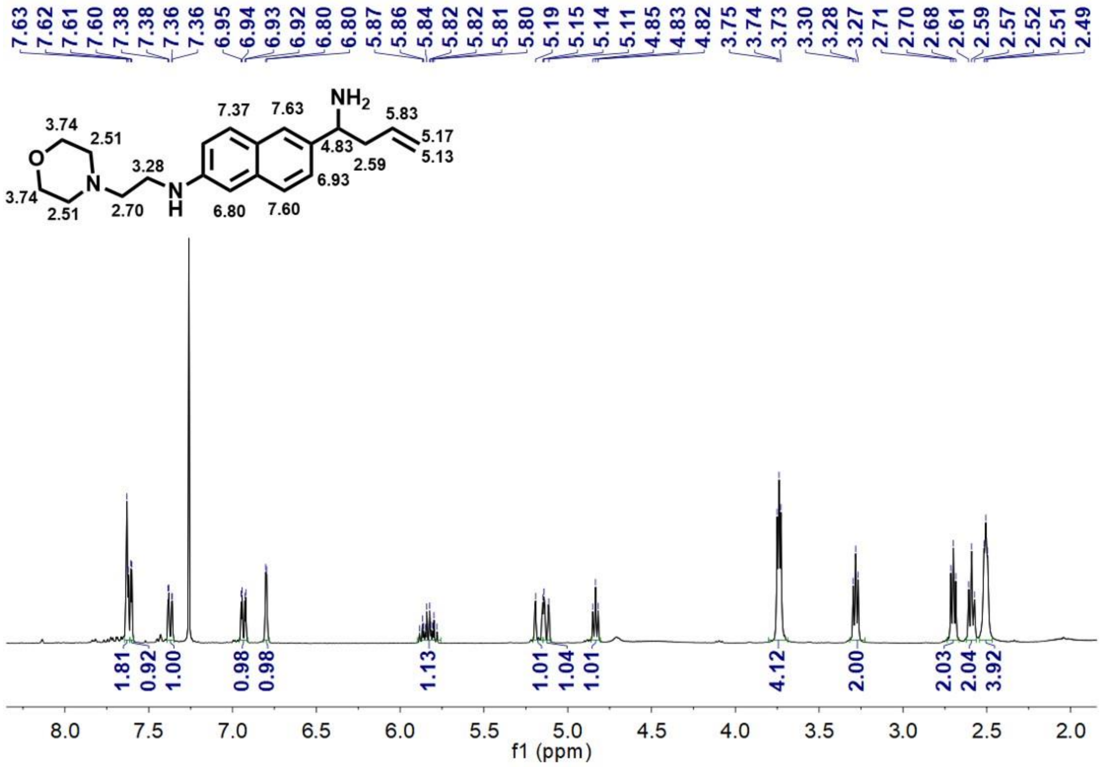

Figure S32. ^{1}H NMR spectrum of compound F(CDCl$_3$).

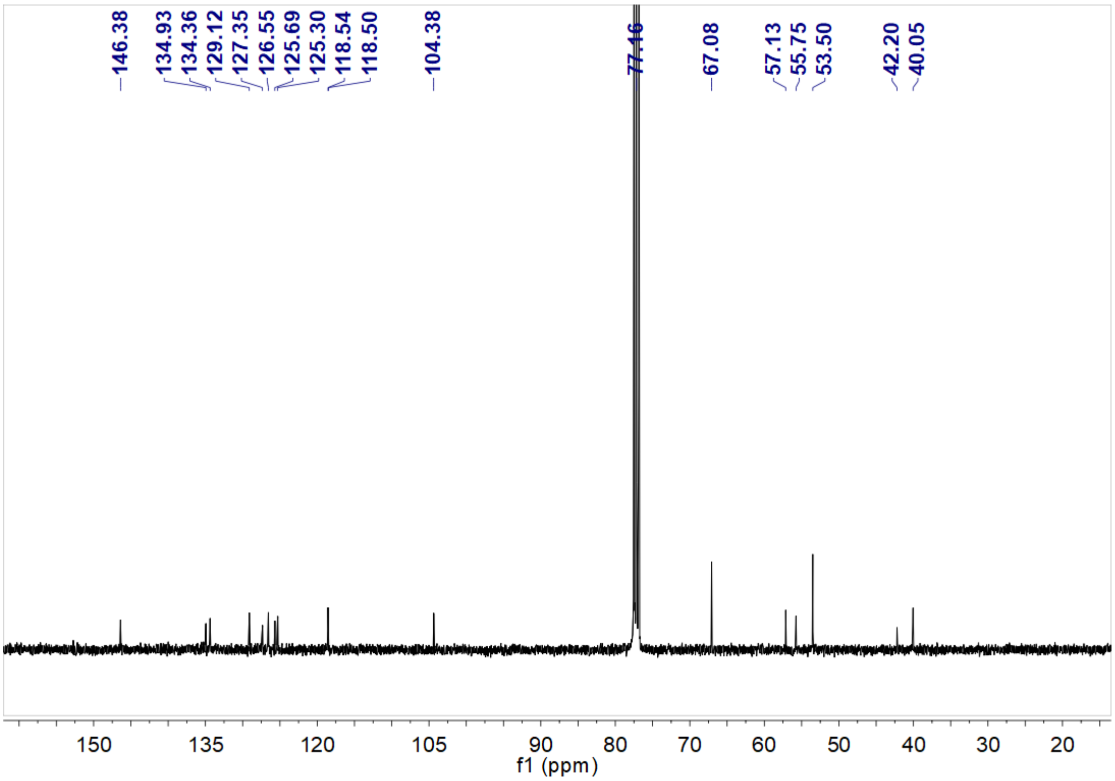

Figure S33. ^{13}C NMR spectrum of compound F (CDCl$_3$).

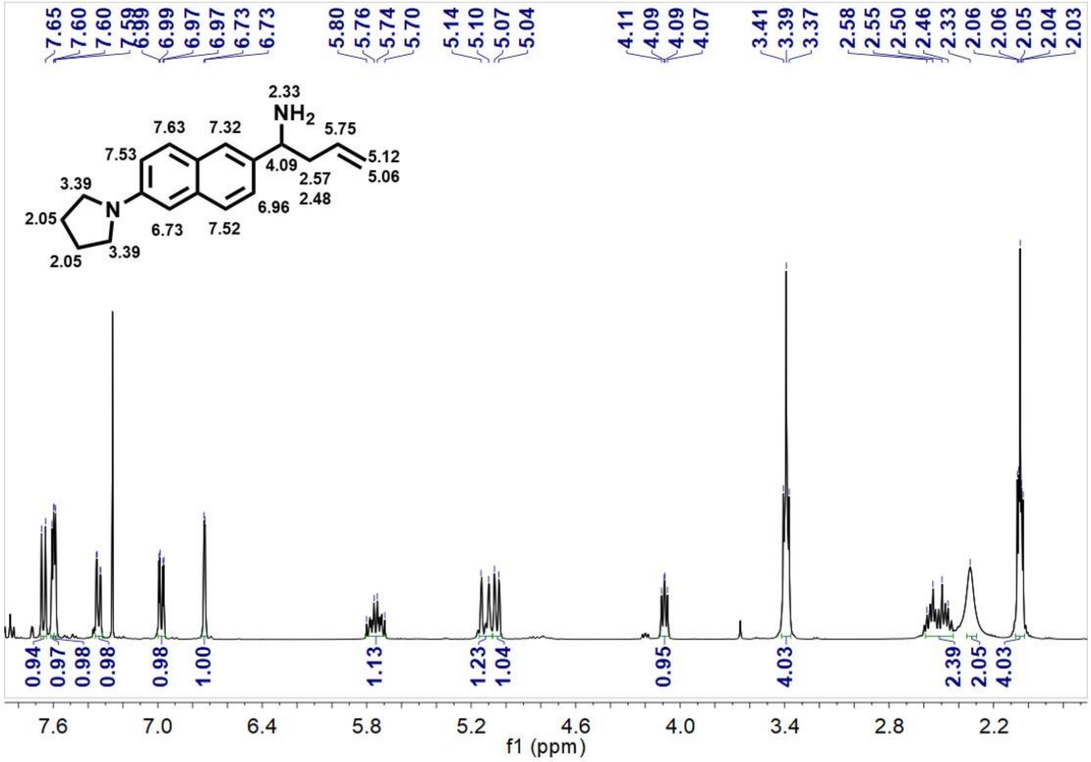

Figure S34. ^{1}H NMR spectrum of **FA-1** ($CDCl_3$).

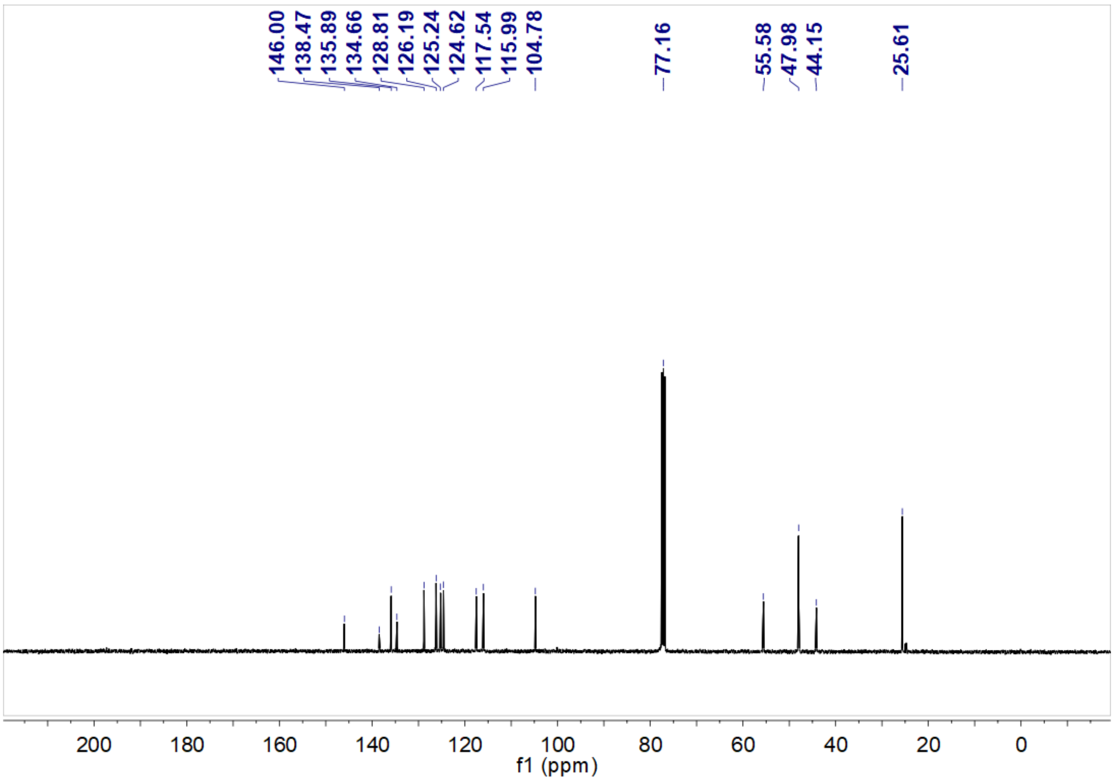

Figure S35. ^{13}C NMR spectrum of **FA-1** ($CDCl_3$).

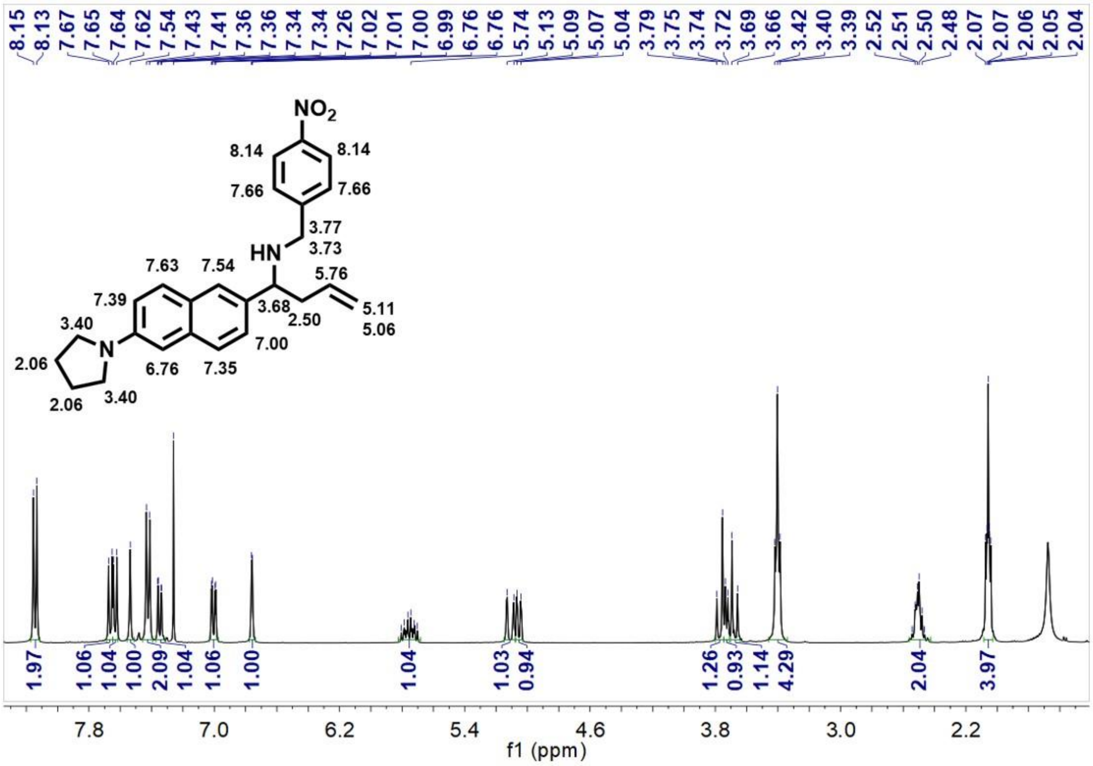

Figure S36. ^{1}H NMR spectrum of **FA-2** ($CDCl_3$).

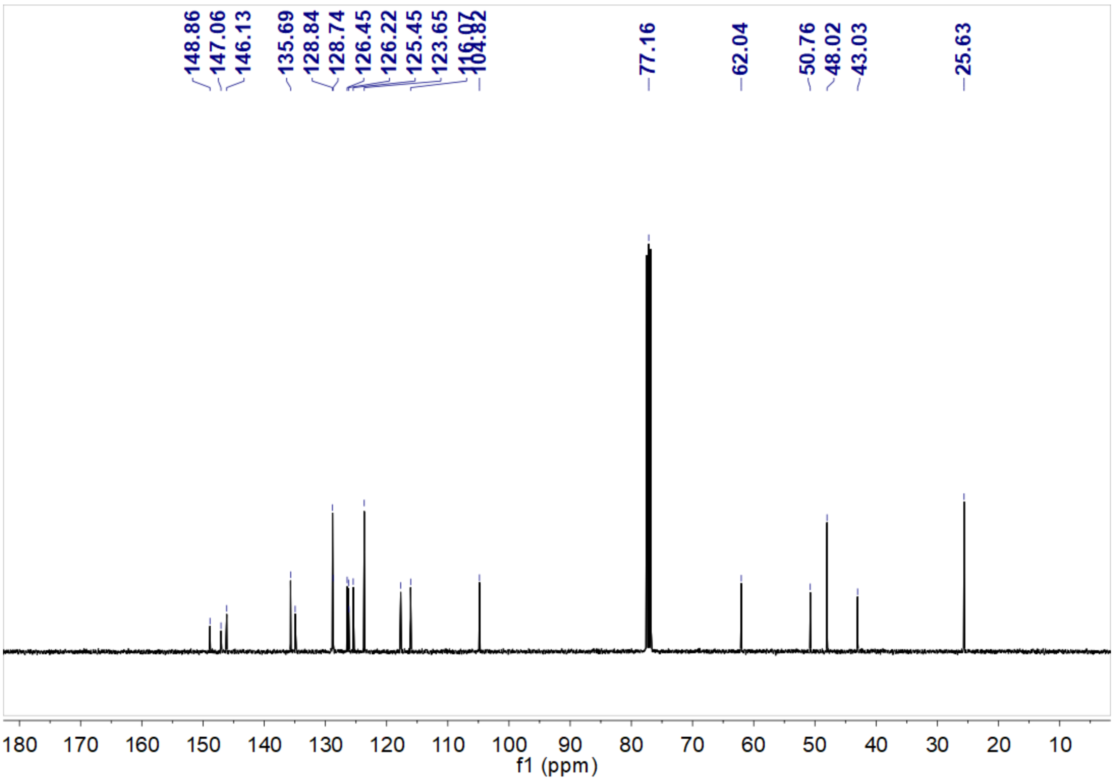

Figure S37. ^{13}C NMR spectrum of **FA-2** ($CDCl_3$).

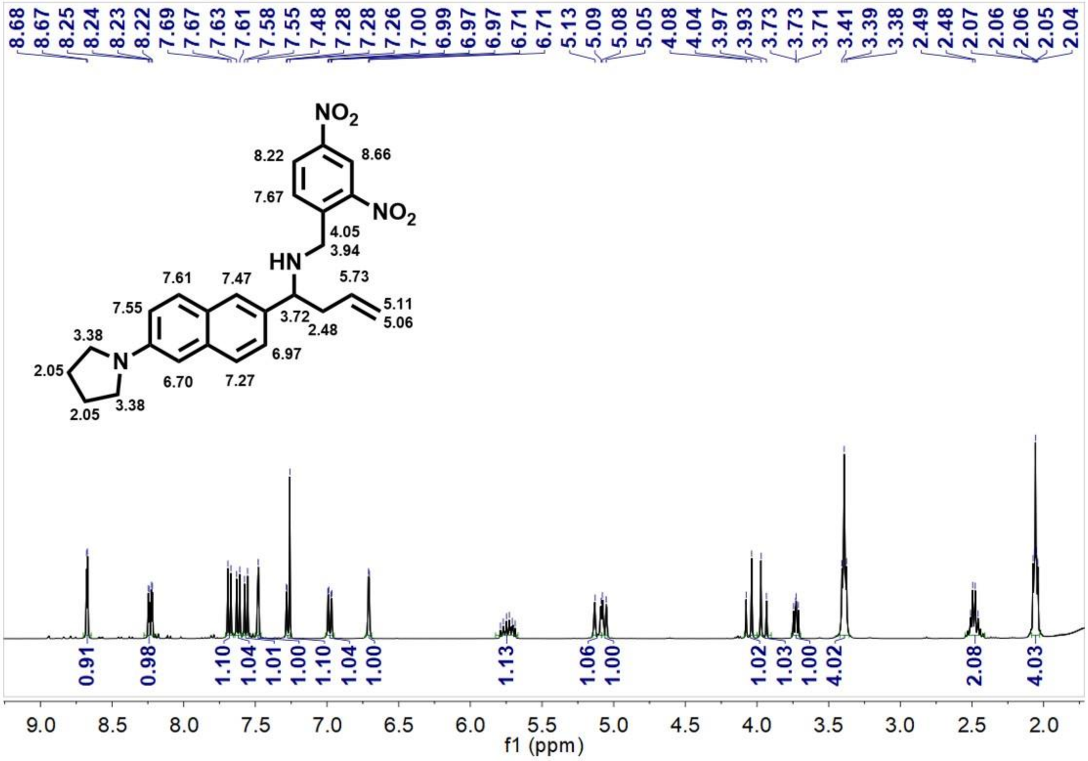

Figure S38. ^{1}H NMR spectrum of **FA-3** ($CDCl_3$).

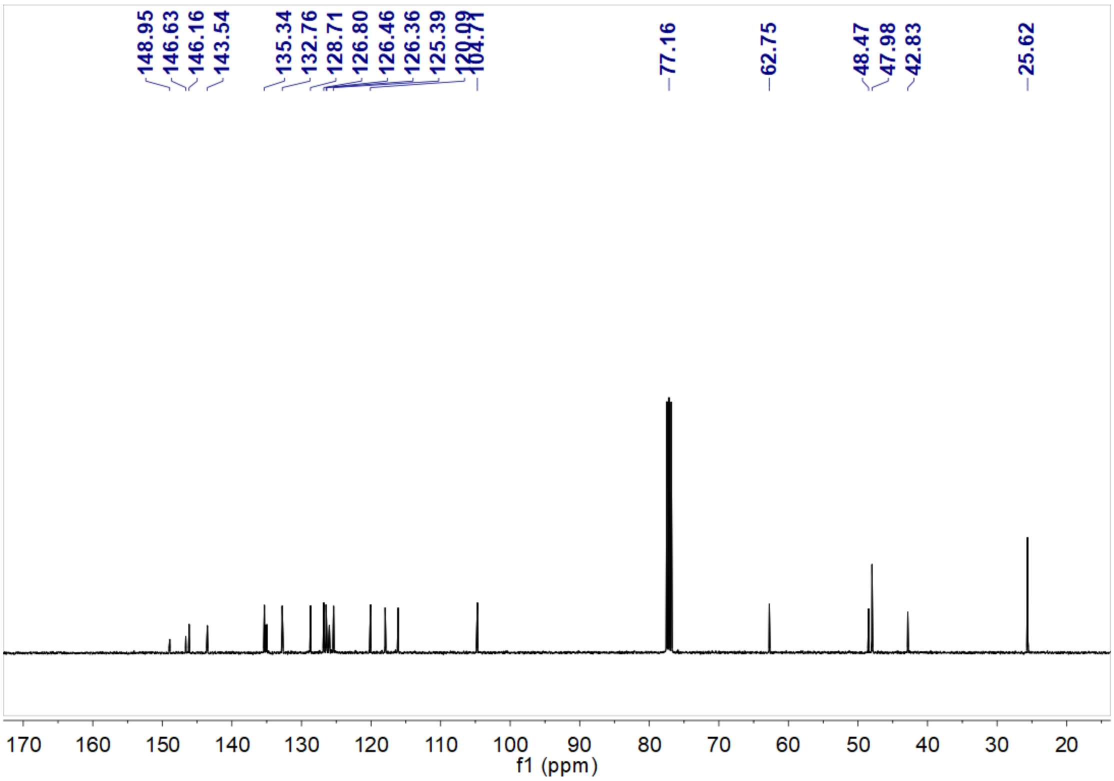

Figure S39. ^{13}C NMR spectrum of **FA-3** ($CDCl_3$).

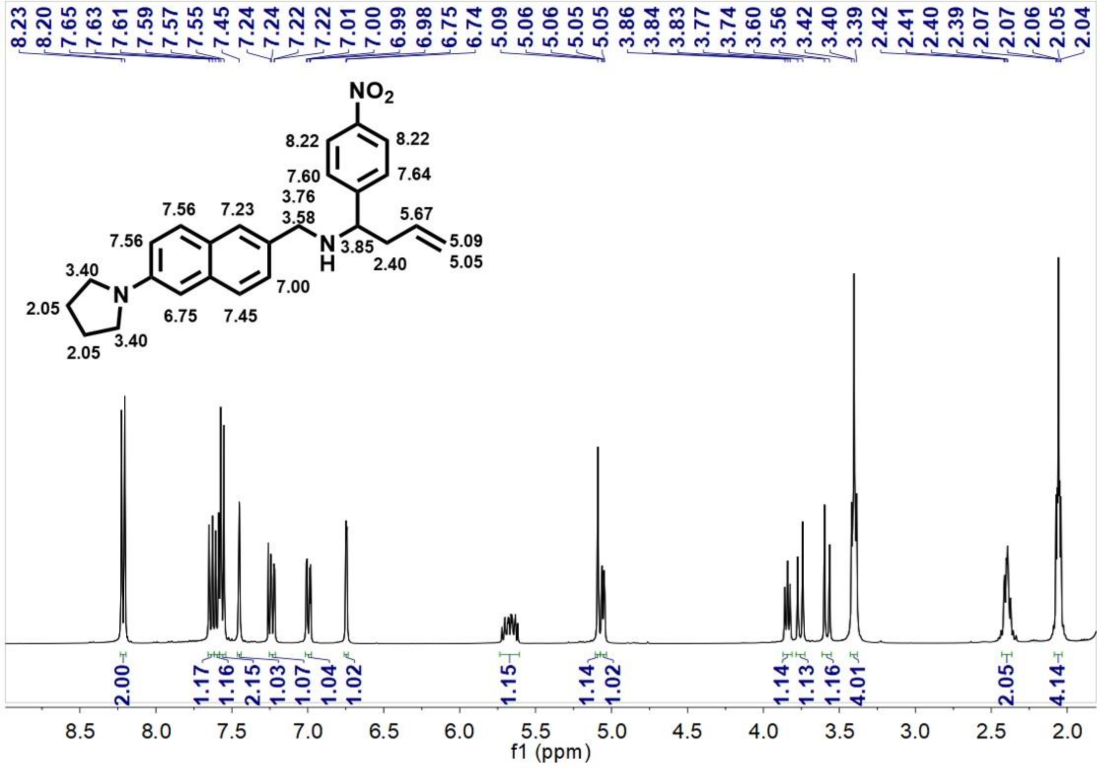

Figure S40. ^{1}H NMR spectrum of **FA-4** ($CDCl_3$).

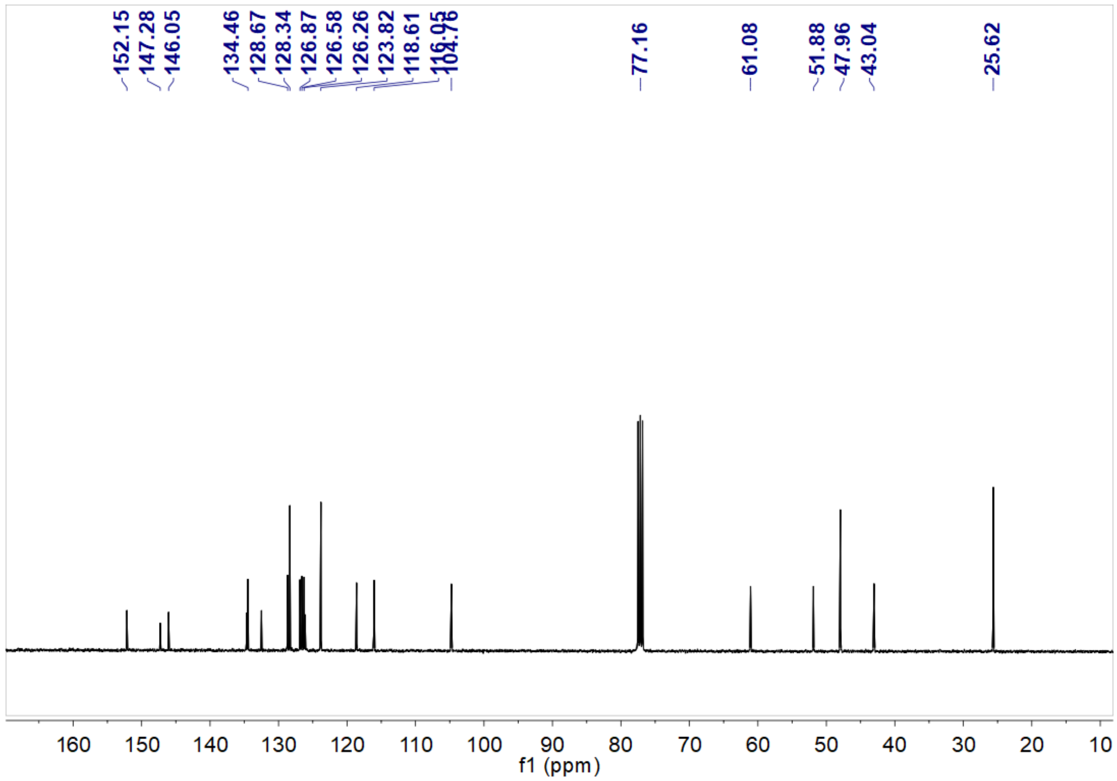

Figure S41. ^{13}C NMR spectrum of **FA-4** ($CDCl_3$).

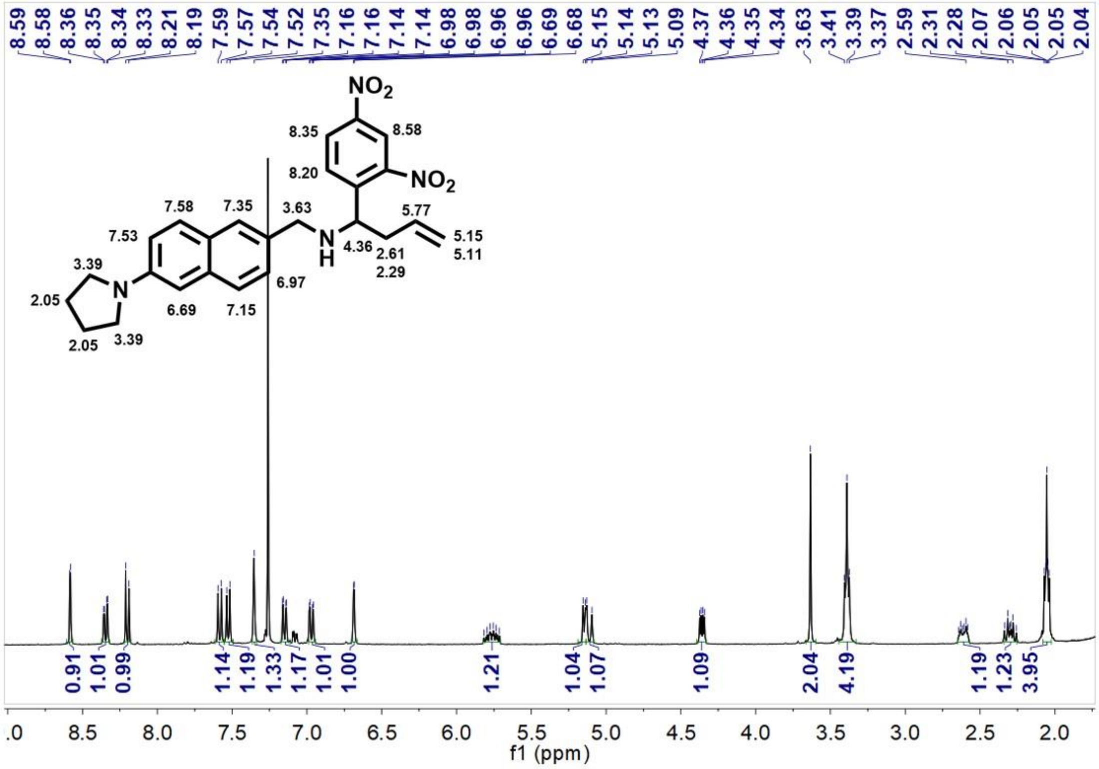

Figure S42. ^{1}H NMR spectrum of **FA-5** ($CDCl_3$).

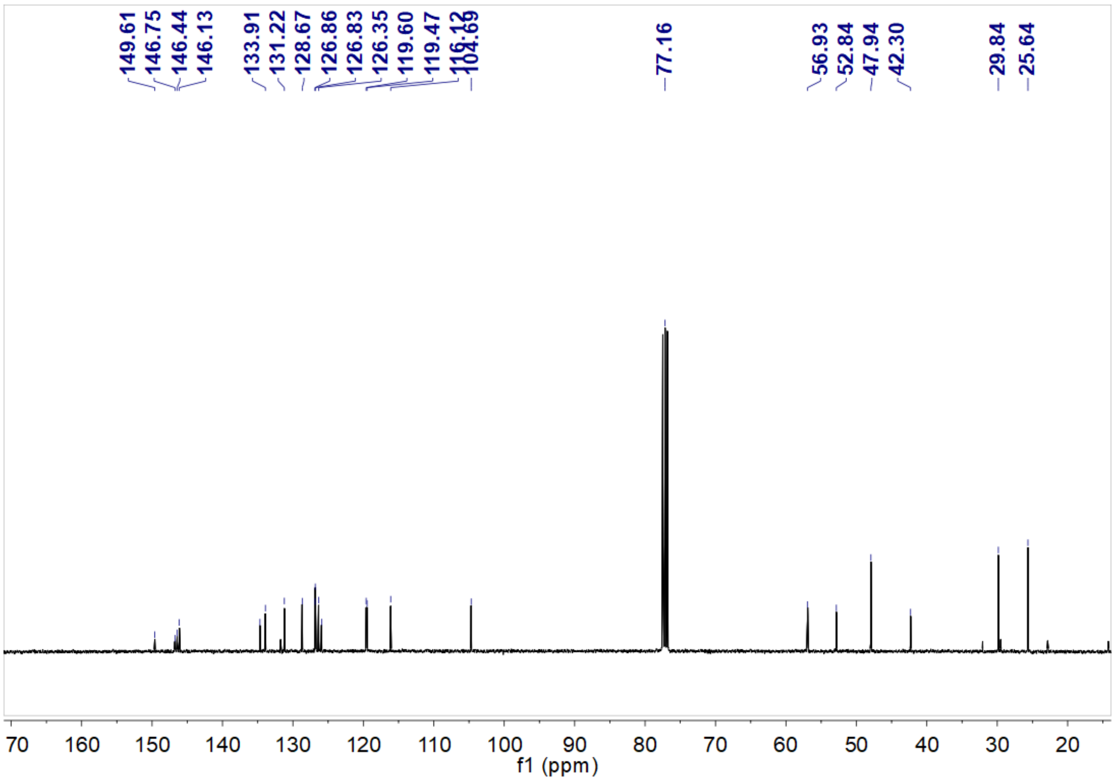

Figure S43. ^{13}C NMR spectrum of **FA-5** ($CDCl_3$).

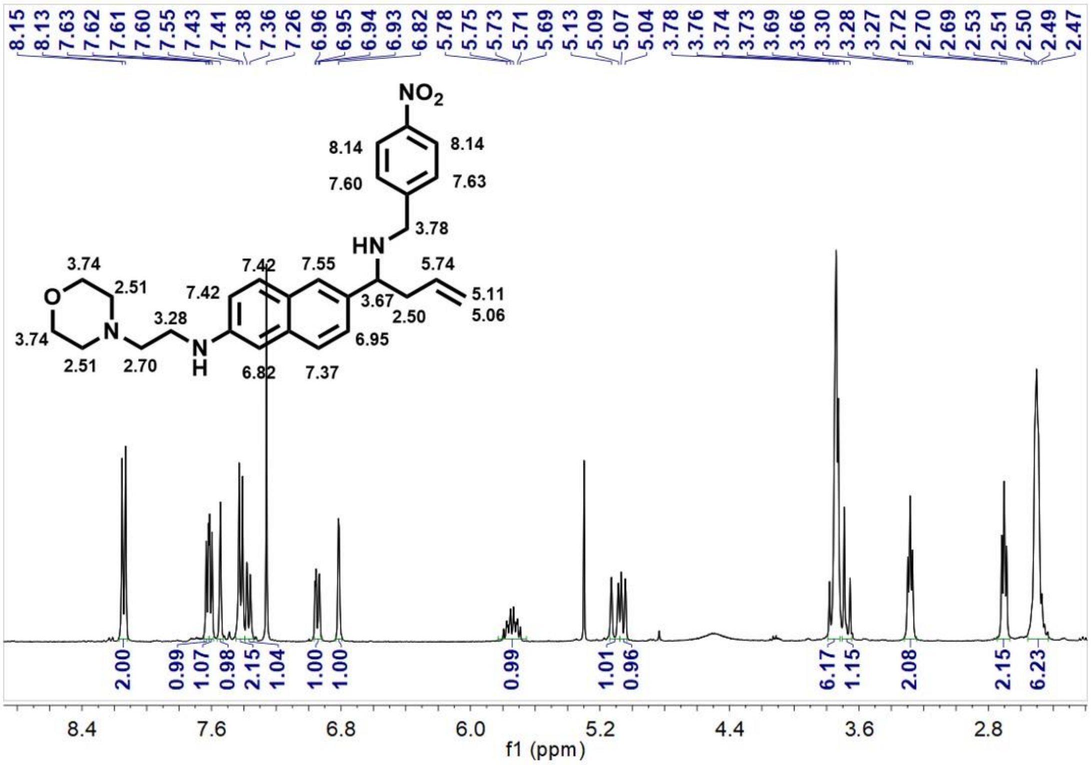

Figure S44. ^{1}H NMR spectrum of **FA-Lyso** ($CDCl_3$).

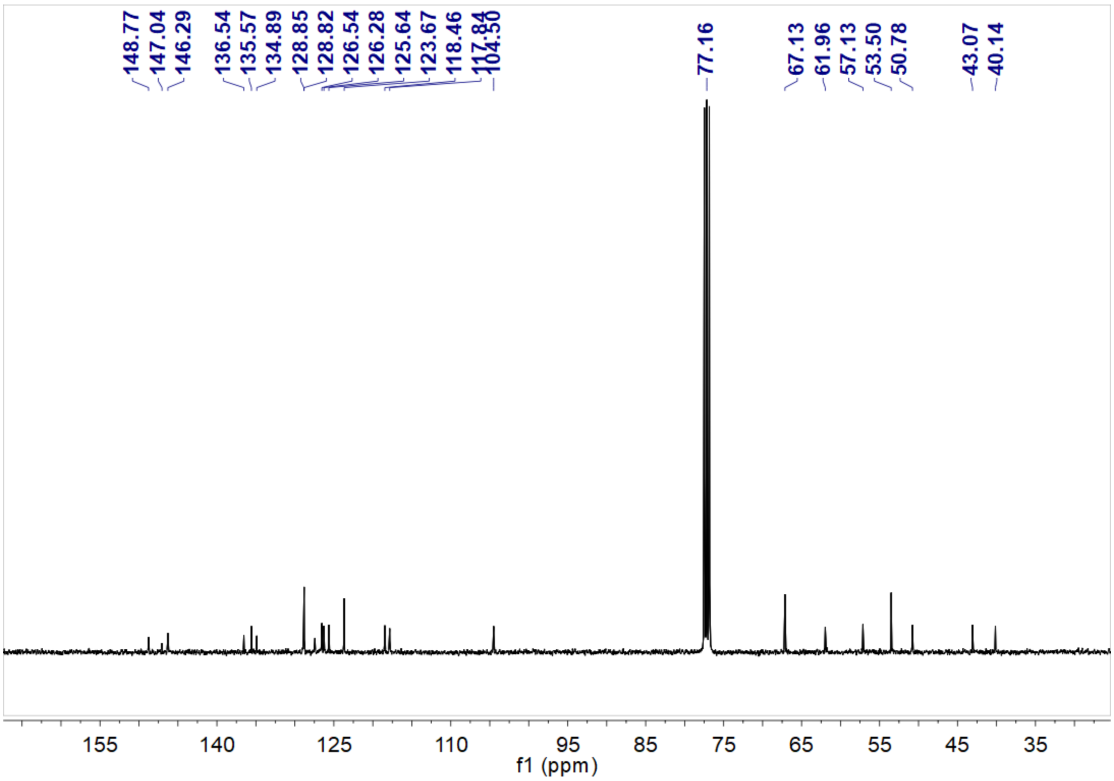

Figure S45. ^{13}C NMR spectrum of **FA-Lyso** ($CDCl_3$).

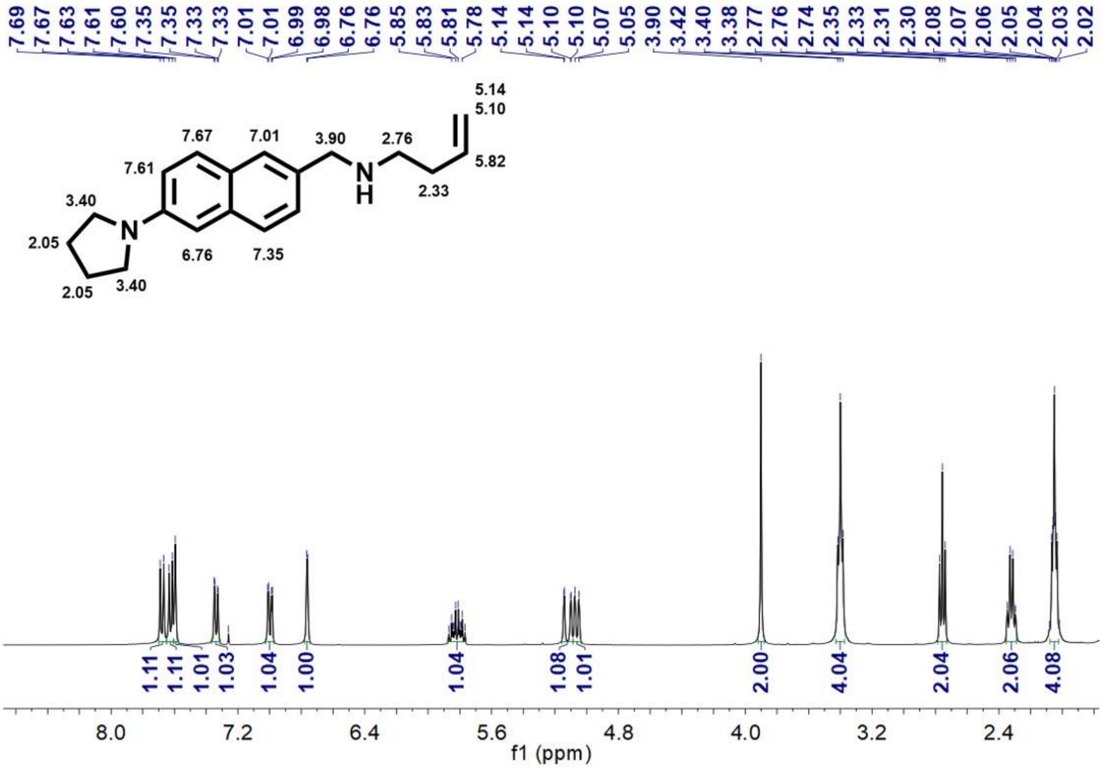

Figure S46. ^{1}H NMR spectrum of **FA-P** ($CDCl_3$).

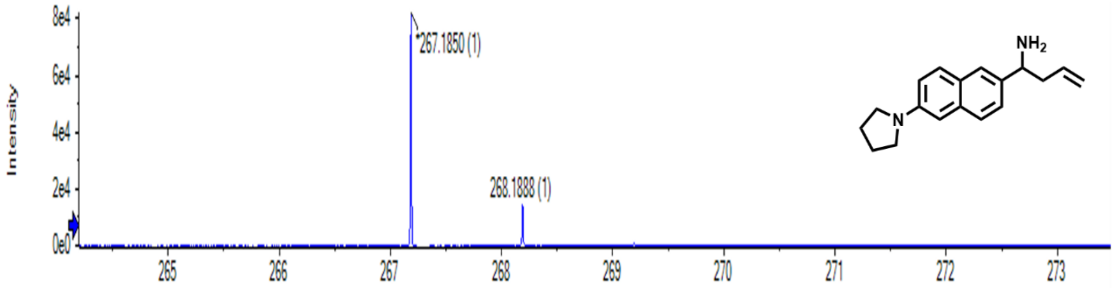

Figure S47. ESI-MS spectrum of **FA-1.**

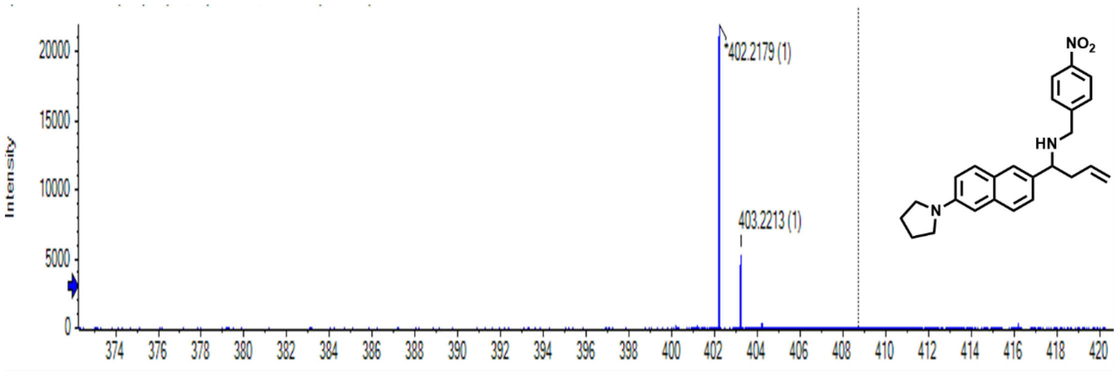

Figure S48. ESI-MS spectrum of **FA-2.**

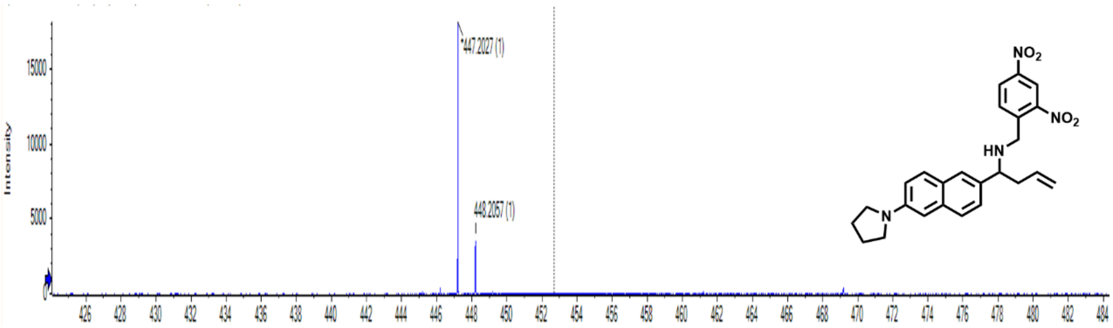

Figure S49. ESI-MS spectrum of **FA-3.**

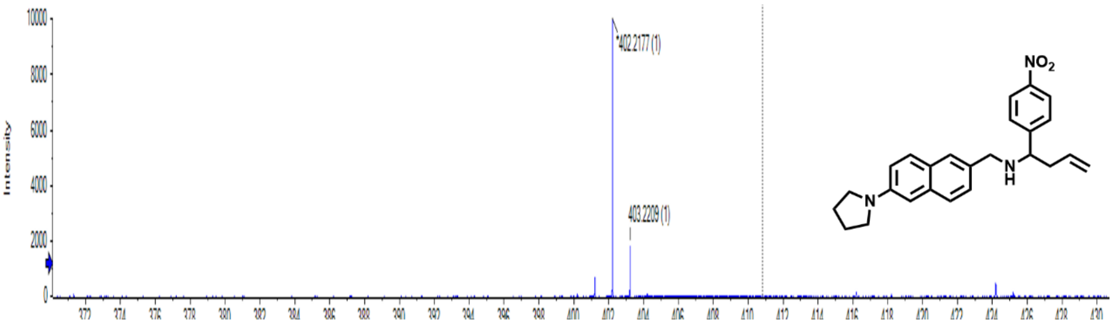

Figure S50. ESI-MS spectrum of **FA-4.**

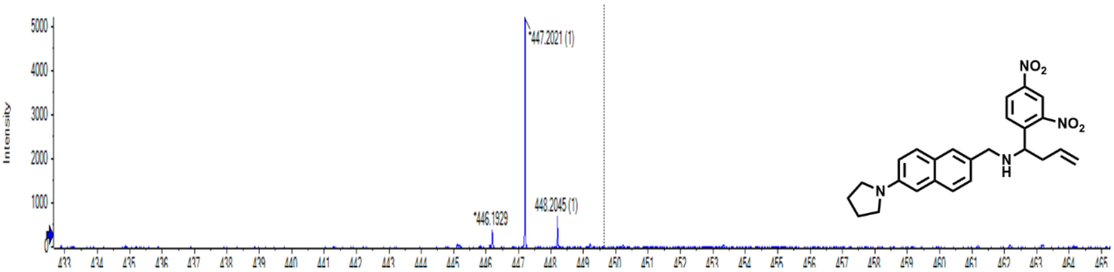

Figure S51. ESI-MS spectrum of **FA-5.**

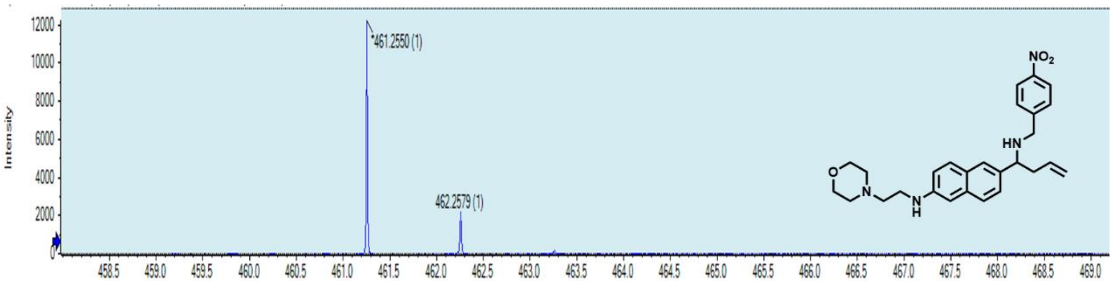

Figure S52. ESI-MS spectrum of FA-Lyso.
